# Fabry disease

**DOI:** 10.1186/1750-1172-5-30

**Published:** 2010-11-22

**Authors:** Dominique P Germain

**Affiliations:** 1 University of Versailles - St Quentin en Yvelines (UVSQ), Faculté de Médecine Paris - Ile de France Ouest (PIFO), 78035 Versailles, France; 2 Division of Medical Genetics, CHU Raymond Poincaré (Assistance Publique - Hôpitaux de Paris), 92380 Garches, France

## Abstract

Fabry disease (FD) is a progressive, X-linked inherited disorder of glycosphingolipid metabolism due to deficient or absent lysosomal **α**-galactosidase A activity. FD is pan-ethnic and the reported annual incidence of 1 in 100,000 may underestimate the true prevalence of the disease. Classically affected hemizygous males, with no residual **α**-galactosidase A activity may display all the characteristic neurological (pain), cutaneous (angiokeratoma), renal (proteinuria, kidney failure), cardiovascular (cardiomyopathy, arrhythmia), cochleo-vestibular and cerebrovascular (transient ischemic attacks, strokes) signs of the disease while heterozygous females have symptoms ranging from very mild to severe. Deficient activity of lysosomal **α**-galactosidase A results in progressive accumulation of globotriaosylceramide within lysosomes, believed to trigger a cascade of cellular events. Demonstration of marked **α**-galactosidase A deficiency is the definitive method for the diagnosis of hemizygous males. Enzyme analysis may occasionnally help to detect heterozygotes but is often inconclusive due to random X-chromosomal inactivation so that molecular testing (genotyping) of females is mandatory. In childhood, other possible causes of pain such as rheumatoid arthritis and 'growing pains' must be ruled out. In adulthood, multiple sclerosis is sometimes considered. Prenatal diagnosis, available by determination of enzyme activity or DNA testing in chorionic villi or cultured amniotic cells is, for ethical reasons, only considered in male fetuses. Pre-implantation diagnosis is possible. The existence of atypical variants and the availability of a specific therapy singularly complicate genetic counseling. A disease-specific therapeutic option - enzyme replacement therapy using recombinant human **α**-galactosidase A - has been recently introduced and its long term outcome is currently still being investigated. Conventional management consists of pain relief with analgesic drugs, nephroprotection (angiotensin converting enzyme inhibitors and angiotensin receptors blockers) and antiarrhythmic agents, whereas dialysis or renal transplantation are available for patients experiencing end-stage renal failure. With age, progressive damage to vital organ systems develops and at some point, organs may start to fail in functioning. End-stage renal disease and life-threatening cardiovascular or cerebrovascular complications limit life-expectancy of untreated males and females with reductions of 20 and 10 years, respectively, as compared to the general population. While there is increasing evidence that long-term enzyme therapy can halt disease progression, the importance of adjunctive therapies should be emphasized and the possibility of developing an oral therapy drives research forward into active site specific chaperones.

## Review

### I - Disease name and synonyms

Fabry disease

Fabry's disease

Anderson-Fabry disease

Alpha-galactosidase A deficiency

Angiokeratoma corporis diffusum

Ceramide trihexosidosis

Ruiter-Pompen-Wyers syndrome

Sweeley-Klionsky disease

### II - Definition

Fabry disease (FD, OMIM 301500) [1,2] is a devastating, progressive inborn error of metabolism with, particularly in the early stages, important roles being played by cellular dysfunction and microvascular pathology induced by lysosomal glycosphingolipid deposition [3]. Absent or deficient activity of lysosomal exoglycohydrolase **α**-galactosidase A (**α**-D-galactoside galactohydrolase, EC 3.2.1.22; **α**-gal A) [4,5] results in progressive accumulation of globotriaosylceramide (Gb_3 _or GL-3; also known as ceramidetrihexoside or CTH) and related glycosphingolipids (galabiosylceramide) within lysosomes which are ubiquitous subcellular organelles [6], in a variety of cell types, including capillary endothelial cells, renal (podocytes, tubular cells, glomerular endothelial, mesangial and intersticial cells), cardiac (cardiomyocytes and fibroblasts) and nerve cells [7]. The primary disease process starts in infancy, or even as early as in the fetal stage of development [8,9]. However, in contrast to many other lysosomal storage diseases [10,11], most patients remain clinically asymptomatic during the very first years of life. In FD, lysosomal storage and cellular dysfunction are believed to trigger a cascade of events including cellular death, compromised energy metabolism [12-14], small vessel injury [15], K(Ca)3.1 channel dysfunction in endothelial cells [16], oxidative stress [17], impaired autophagosome maturation [18], tissue ischemia and, importantly, development of irreversible cardiac [19-21] and renal [22] tissue fibrosis. The first clinical symptoms interfering with the child's well-being and performance arise in childhood, typically between the ages of 3 and 10 years, and generally a few years later in girls than in boys [23,24]. With age, progressive damage to vital organ systems develops in both genders [24] leading to organ failure. End-stage renal disease and life-threatening cardiovascular or cerebrovascular complications limit life-expectancy [25-27].

FD has long been regarded as an *adult *disease with most, if not all, affected males developing a "classic" phenotype. Later on, the sub-classifications "cardiac variant" [28,29] and "renal variant" [30] were introduced for patients with predominant or exclusive cardiac or renal involvement, respectively. Female heterozygotes were erroneously described as "carriers of the defective gene" more or less safeguarded against developing disease manifestations and symptoms. However, evolving knowledge about the natural course of disease suggests that it is more appropriate to describe FD as a disease with a wide spectrum of heterogeneously progressive clinical phenotypes. This spectrum ranges from the "classic" severe phenotype in males to a seemingly asymptomatic disease course occasionally observed in females, with a variety of clinical presentations inbetween. Indeed, most female heterozygotes develop symptoms due to yet undetermined mechanisms [24,31,32] and a high percentage of females develop vital organ involvement including the kidneys, heart and/or brain about a decade later than males [24].

### III - Epidemiology

FD belongs to a group of at least 50 genetically distinct, biochemically related lysosomal storage disorders. Each disorder is caused by an inborn error of metabolism due to a monogenetic defect specifically resulting in the deficiency of lysosomal enzyme(s). FD is pan-ethnic, but due to its rarity, determining an accurate disease frequency is difficult. Reported incidences, ranging from 1 in 476,000 [33] to 1 in 117,000 [34] in the general population, may largely underestimate the true prevalence (Table 1). Newborn screening initiatives have found an unexpectedly high prevalence of the disease, as high as 1 in ~3,100 newborns in Italy [35] and have identified a surprisingly high frequency of newborn males with FD (approximately 1 in 1,500) in Taiwan, 86% having the IVS4+919G > A cryptic splice mutation previously found in later-onset cardiac phenotype patients [36] (Table 1). The intronic IVS4+919G > A mutation was also found in a number of Taiwan Chinese adult patients with idiopathic hypertrophic cardiomyopathy [37].

**Table 1:** Studies of prevalence of Fabry disease

Methods	Source	Ascertainment period	Total number of cases	No. per 100000	Country and reference
Birth prevalence (number of postnatal plus prenatal enzymatic diagnoses divided by number of births)	Two centres holding all enzymatic analyses in Australia	1980-1996	36	0.85	Australia [34]

Birth prevalence (number of cases born within a certain period divided by total number of live births in the same period)	All the laboratories making pre- and postnatal diagnoses of LSDs in The Netherlands	1970-1996	27	0.21	The Netherlands [33]

Prevalence of obligate carriers	By family history, from the UK AFD register	1980-1995	60	0.29	UK (females only) [26]

Prevalence	Records from regional genetic units and enzyme reference laboratories; records from individual doctors	1980-1995	98	0.27	UK (males only) [425]

Birth prevalence (number of cases born within a certain time period divided by total number of live births in the same period)	Two main reference centres for diagnosis of sphingolipidoses by enzyme analysis of patients under 5 years suspected of LSD	1997-2002	1	0.015	Turkey [426]

Birth prevalence (number of postnatal plus prenatal enzymatic diagnoses divided by number of live births) in north Portugal	One centre providing all pre- and postnatal diagnoses of LSDs in Portugal	1982-2001	1	0.12	North Portugal [427]

Neonatal screening	Northern Italy	2004-2006	12	30	Italy [35]

Neonatal screening	Taiwan	2006-2008	73	80	Taiwan [36]

### IV - Clinical description

#### A. Early signs and symptoms: Fabry disease at the pediatric age

Early neural damage primarily involves small nerve fibers of the peripheral somatic [38] and autonomic nerve systems [39] with onset of related symptoms generally occurring at an earlier age in boys than in girls [23,40-42]. Pain is experienced by 60-80% of classically affected boys and girls [23,43] and is one of the earliest symptoms of FD. Two types of pain have been described: episodic crises ("Fabry crises") characterized by agonizing burning pain originating in the extremities and radiating inwards to the limbs and other parts of the body, and chronic pain characterized by burning and tingling paraesthesias [44]. Fabry crises may be precipitated by fever, exercise, fatigue, stress, and rapid changes in temperature [45]. When the crises are triggered or accompanied by fever, patients usually also have an elevated erythrocyte sedimentation rate. As a result of their pain, patients with FD have a greatly diminished quality of life [46,47]. Other possible causes of pain that must be ruled out are rheumatoid arthritis, rheumatic fever, Raynaud's disease, systemic lupus erythematosus (SLE) and 'growing pains' (a frequent misdiagnosis in children with FD) (Table 2). Pain may wane in adulthood and it is important to search for a medical history of acroparesthesia in childhood during the first examination of a newly diagnosed adult patient [48].

**Table 2:** Early signs and symptoms of Fabry disease

Organ system	Sign/Symptom
**Nervous system**	Acroparesthesias
	Nerve deafness
	Heat intolerance
	Hearing loss, tinnitus

**Gastrointestinal tract**	Nausea, vomiting, diarrhoa
	Postprandial bloating and pain, early satiety
	Difficulty gaining weight

**Skin**	Angiokeratomas
	Hypohidrosis

**Eyes**	Corneal and lenticular opacities
	Vasculopathy (retina, conjunctiva)

**Kidneys**	Microalbuminuria, proteinuria
	Impaired concentration ability
	Hyperfiltration
	Increased urinary Gb3 excretion

**Heart**	Impaired heart rate variability
	Arrhythmias
	ECG abnormalities (shortened PR interval)
	Mild valvular insufficiency

Other early-onset signs appearing in childhood will usually remain present during adulthood and, among them, gastrointestinal involvement is a common, but under-appreciated, manifestation of FD [49]. Patients may complain of abdominal pain (often after eating), diarrhea, nausea, and vomiting, which are a significant cause of anorexia [50]. These gastrointestinal symptoms may be related to the deposition of Gb_3 _in the autonomic ganglia of the bowel and mesenteric blood vessels [51]. Diarrhea-predominant irritable bowel syndrome (IBS) is a differential diagnosis [50].

Absence of sweating (anhidrosis) [52] or a decreased ability to sweat (hypohidrosis) [53] with decreased skin impedance [54] is a significant problem for patients and can cause heat [55] and exercise intolerance [51,56].

**Figure 1:**
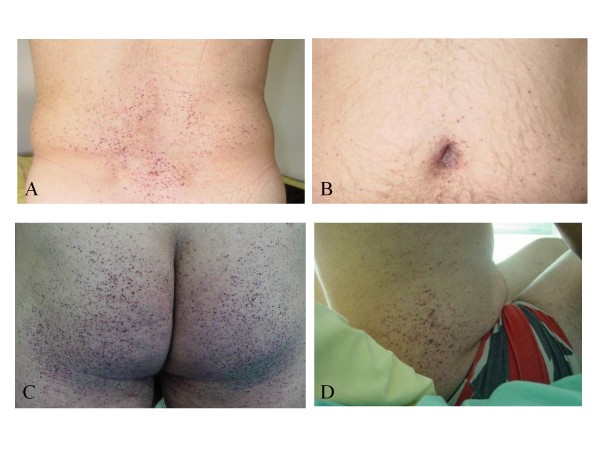
**Angiokeratoma**: the angiokeratoma are small, raised, dark-red spots that increase in number and size with age and can occur singly or in clusters. They are typically found on the lower back (A), buttocks (C), groin, flanks (D) and upper thighs but their distribution may be restricted to a limited area, such as the umbilicus (B).

**Figure 2:**
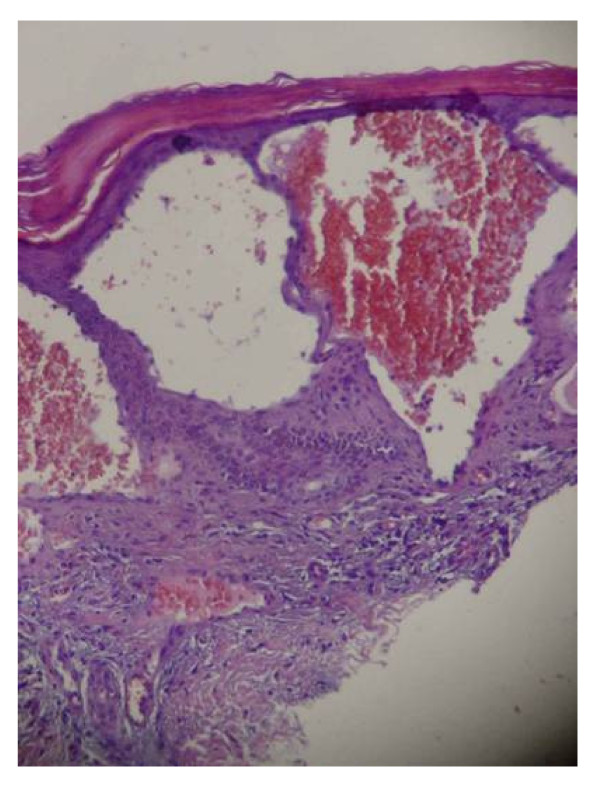
**Skin biopsy (light microscopy)**: histologically, the typical skin lesion is a small superficial angioma caused by cumulative damage of the vascular cells of the dermis with vessel dilation. Courtesy: Dr Juan M. POLITEI, Buenos Aires, Argentina.

The most visible early clinical feature of FD is angiokeratoma (skin lesions) and clusters of small reddish purple, raised skin lesions (Figure 1) are typically found on the buttocks, groin, umbilicus and upper thighs, but also sometimes on mucosal areas, such as the mouth. Histologically, the skin lesions are small superficial angiomas caused by cumulative damage of the vascular endothelial cells of the skin with vessel dilatation in the dermis (Figure 2) that increase in number and size with age and can occur singly or in groups [53,56,57]. Telangiectasia [53,55] and subcutaneous edema [58] have also been reported.

Corneal changes ("*cornea verticillata*"), rarely of visual significance and readily detectable by slit lamp examination, are frequently encountered. Retinal vessel tortuosity may be observed.

Tinnitus may be an early symptom and hearing loss has been reported in children [59].

Chronic fatigue and difficulty gaining weight may also frequently occur, particularly during adolescence. High-flow priapism can also be observed in young boys affected with FD.

Despite the absence of major organ dysfunction, these symptoms, individually or in combination, may cause significant morbidity limiting the child's physical, school and social performances [60]. Early signs and symptoms of FD are presented in Table 2.

Early signs of cardiac and cerebrovascular abnormalities may be present during adolescence in both genders. Signs of involvement of the sinus node and conduction system (e.g. shortened PR interval, arrhythmias, impaired heart rate variability, and mild valvular insufficiency) have been demonstrated [61]. Although rare, evidence of microvascular ischemic brain involvement on magnetic resonance imaging (MRI) may be detectable at young ages [62].

The natural course of Fabry nephropathy in children or adolescent patients is still largely not understood. Signs indicative of early, insidiously progressing renal damage include microalbuminuria and proteinuria developing as early as in the second decade of life [63-65]. Histologic, potentially irreversible changes to glomeruli, interstitial tubules and vascular structures before the first appearance of microalbuminuria can be observed in renal biopsy specimens from children [65]. Podocyte foot process effacement has been reported and indicates focal segmental glomerulosclerosis. A decline in glomerular filtration rate (GFR) is uncommon at pediatric ages but may be seen as early as adolescence [56,66]. Studies on renal function in children with FD have mainly been done using estimated creatinine-based GFR. The widely used original Schwartz formula [67] substantially overestimates GFR with a low accuracy, whereas the new abbreviated Schwartz formula [68] shows relatively good performances with a mean GFR overestimation of 5.3 ml/min/1.73 m^2^, being only slightly superior to the Counahan-Barratt formula [69]. The new abbreviated Schwartz formula should replace the original Schwartz formula in the routine follow-up of children with FD [70]. The current creatinine-based GFR formulas are all hampered by low accuracy in the "creatinine-blind" GFR range. Supplemental measured GFR is, therefore, recommended in patients where changes in GFR have potential impact on important treatment regimens [70].

#### B. Kidney involvement

**Figure 3:**
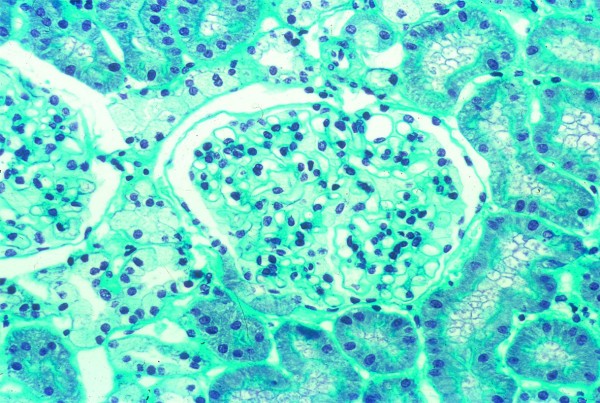
**Kidney biopsy (light microscopy)**: low power view of a glomerulus on a core needle biopsy in Fabry disease, × 320. Courtesy Pr Marie-Claire GUBLER, Paris, France

**Figure 4:**
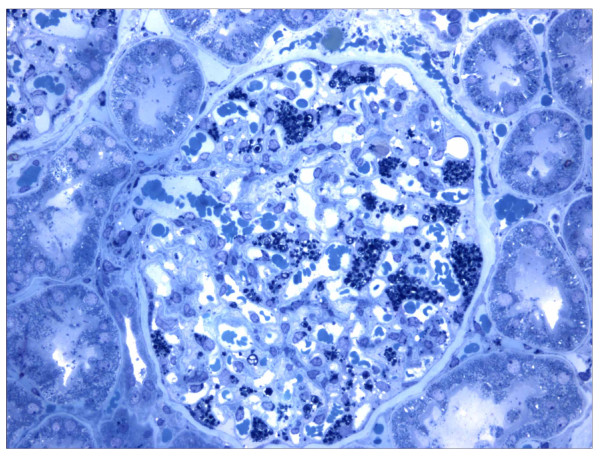
**Kidney biopsy (light microscopy)**: the purple stain is on the podocytes where there is the most prominent collection of Gb_3 _in the kidney. Courtesy Pr Laura BARISONI, New-York University, New York, USA.

**Figure 5:**
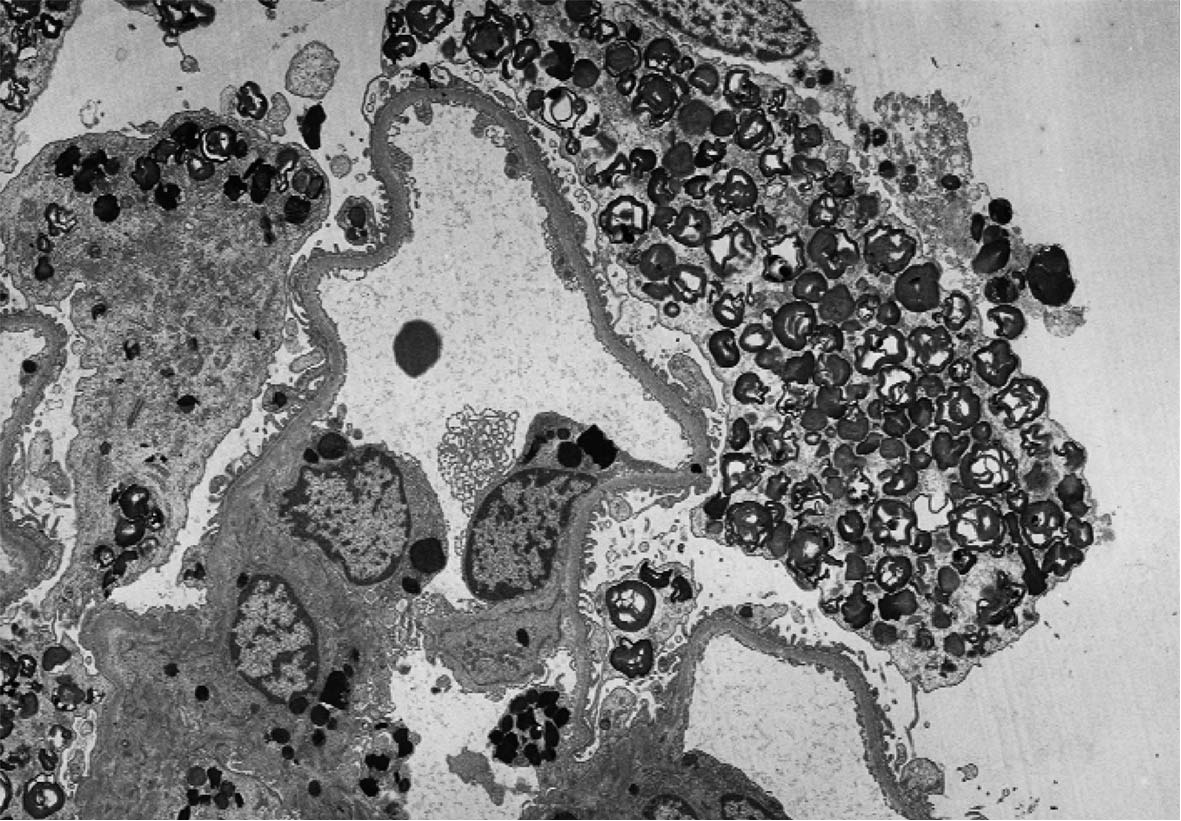
**Kidney biopsy**: electron microscopy shows massive storage of glycosphingolipids in the lysosomes of podocytes. Courtesy: Pr Marie-Claire GUBLER, Paris, France.

**Figure 6:**
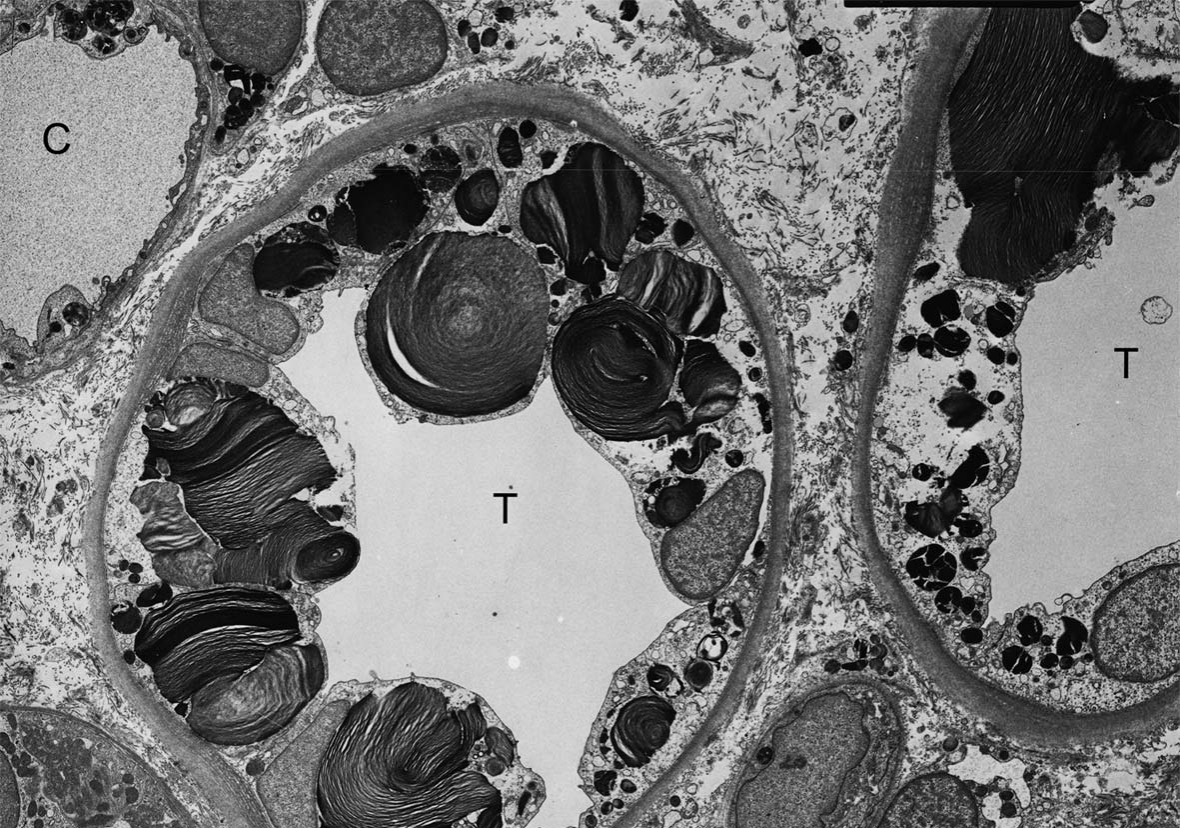
**Kidney biopsy (electron microscopy)**: glycosphingolipid inclusions of various size and shape are seen in the cells of distal tubules of the kidney in Fabry disease.

**Figure 7:**
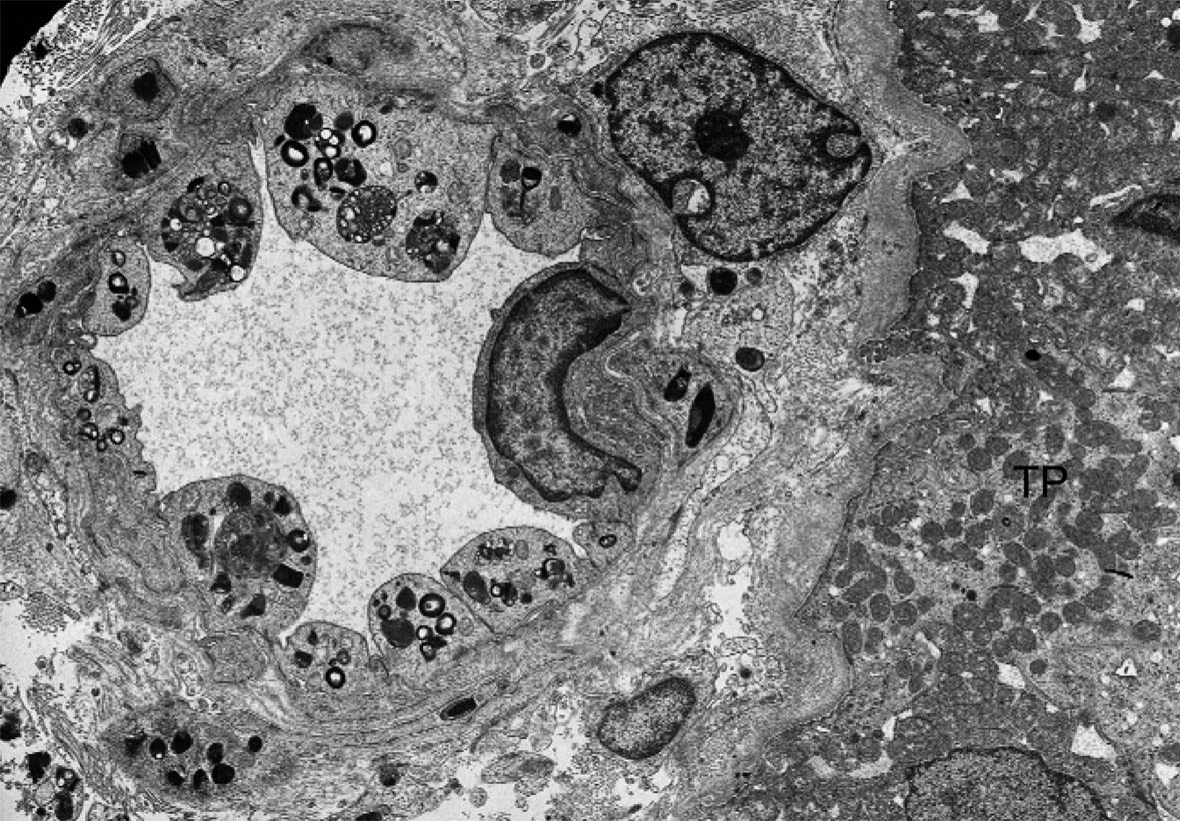
**Kidney biopsy (electron microscopy)**: glycolipid inclusions in the endothelial and smooth muscle cells of a renal arteriole. No storage can be seen in the proximal tubule (TP), × 8200. Courtesy: Pr Marie-Claire GUBLER, Paris, France.

Like most aspects of the disease, renal pathology increases in severity with age. In classically affected Fabry patients, renal lesions result from Gb_3 _deposition in the glomerular endothelial, mesangial, intersticial cells and in podocytes (Figures 3 and 4), which are terminally-differenciated epithelial cells that accumulate numerous myelin-like inclusions in their lysosomes (Figure 5). Podocyte foot process effacement has been described. Glycosphingolipid storage also occur in the epithelium of the loop of Henle and the distal tubules (Figure 6), and in the endothelial and smooth muscle cells of the renal arterioles (Figure 7) [63,71].

Renal impairment often begins with microalbuminuria and proteinuria in the 2^nd ^to 3^rd ^decade of life which, like in diabetic nephropathy, are believed to directly contribute to the progression of the Fabry nephropathy. With advancing age, proteinuria worsens [72]. Isosthenuria accompanied by alterations in tubular reabsorption, secretion and excretion develop. Initially, glomerular compensation (hyperfiltration) may mask impairment of renal function but, once a critical number of nephrons have been damaged, renal function will progressively decline. Gradual deterioration of renal function and development of azotemia usually occur in the third to fifth decades of life [73]. At this stage, fibrosis, sclerosis, and tubular atrophy dominate the disease activity portending end-stage renal disease that generally occurs in males in the 4th to 5th decade of life [25,74]. The nephrological aspects of FD are major contributors to the morbidity and mortality associated with the disorder. Progression to end-stage renal failure is the primary cause of death in male patients with untreated FD and death most often results from uremia, unless chronic hemodialysis or renal transplantation is undertaken [25].

The evaluations of kidney function that should be carried out in every patient include serum creatinin, cystatin C, estimates of GFR, total protein, (micro) albumin excretion and urinary sodium excretion. In early stages of kidney involvement, quantitative estimates of GFR are necessary [75]. The utility of "spot" urine protein/creatinine ratios and estimated GFR with the modification of diet with renal disease (MDRD) equation has been established. Assessment of proteinuria and GFR can be used for the staging of chronic kidney disease (CKD), as described in the Kidney Disease Outcomes Quality Initiative (K/DOQI CKD) guidelines [51]. Kidney biopsies may be useful as a baseline assessment and in patients with atypical presentations, including a repeat kidney biopsy when the disease is progressing despite therapy [71].

Urinary protein excretion is strongly associated with renal disease progression in men and women with Fabry disease [76,77].

#### C. Cardiac involvement

Cardiac symptoms including left ventricular hypertrophy, arrhythmia, angina and dyspnea are reported in approximately 40-60% of patients with FD [25,78-81]. Arrhythmias and impaired heart rate variability arise from involvement of the sinus node, conduction system and imbalance between sympathetic and parasympathetic tone. Diastolic dysfunction and concentric left ventricular hypertrophy, which is typically non-obstructive, are important features, with men generally more severely affected than women. Myocardial ischemia and infarction may result from compromised function of the coronary vascular bed [82]. With age, progressive myocardial fibrosis develops with both intersticial and replacement fibrosis [21,83]. Replacement fibrosis almost always starts in the posterior-lateral wall and in the mid-myocardium. In end-stage patients, transmural replacement fibrosis gradually reduces cardiac function to the stage of congestive heart failure [19,84-86]. Malignant arrhythmias are responsible for a number of cardiac deaths in patients affected with FD [81,86,87].

##### Left ventricular structural changes

**Figure 8:**
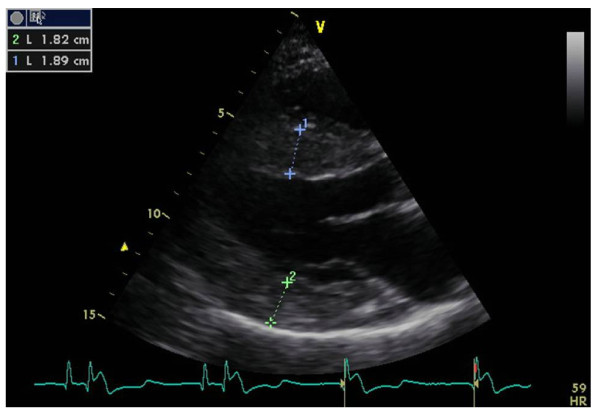
**Echocardiography**: parasternal long axis showing diffuse left ventricular hypertrophy with increased septal thickness. Courtesy: Pr Albert A. HAGEGE, University René Descartes, Paris, France.

**Figure 9:**
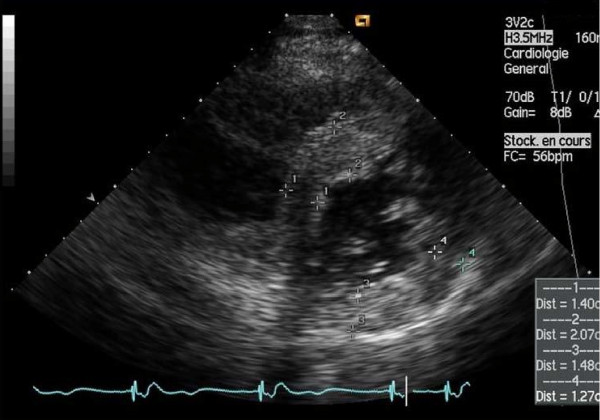
**Echocardiography**: parasternal short axis showing left ventricular hypertrophy. Courtesy: Pr Albert A. HAGEGE, Université René Descartes, Paris, France.

**Figure 10:**
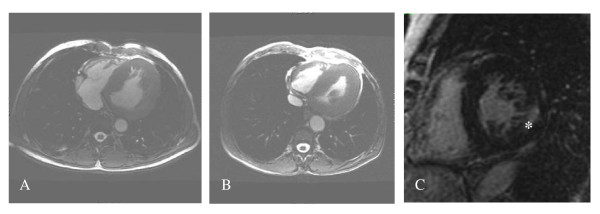
**Cardiac MRI for the assessment of left ventricular hypertrophy and fibrosis**: A: Left ventricular hypertrophy in a 51-year-old male patient with cerebrovascular involvement and end stage renal disease (dialysis). B: Hypertrophic cardiomyopathy in a 56-year-old male patient with arrythmya, leukoareiosis and kidney transplant. C: Late enhancement after gadolinium in a 63-year-old female patient with end stage renal disease (dialysis).

**Figure 11:**
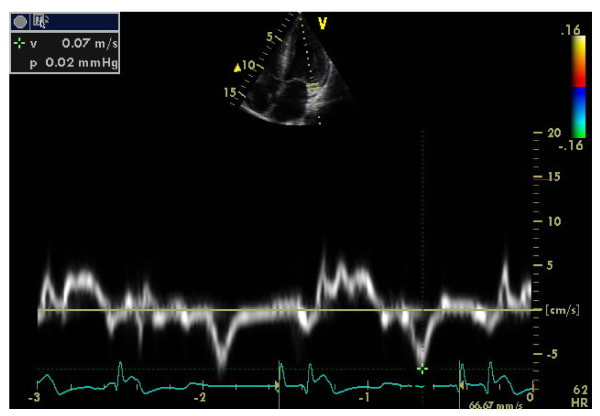
**Tissue Doppler of the mitral annulus: near normal systolic function.** Courtesy: Pr Albert A. HAGEGE, University René Descartes, Paris, France.

**Figure 12:**
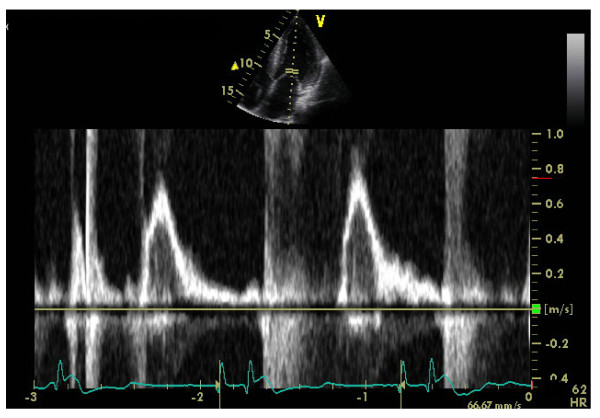
**Doppler: near normal systolic function (same patient as in figure 10).** Courtesy: Pr Albert A. HAGEGE, University René Descartes, Paris, France.

Left ventricular (LV) structural abnormalities are frequent in patients with FD and can be demonstrated using echocardiography (Figures 8 and 9) or cardiac MRI (Figure 10) [19,78-80]. It is particularly important to measure the septum thickness since the posterior wall may become thinner with age due to replacement fibrosis. Concentric hypertrophy has been reported as the most common structural change [78]. Despite these structural changes, however, systolic function appears to be largely preserved when assessed with conventional measurements [19,78-80,84,88]. The cardiomyopathy of FD is characterized by reduced myocardial contraction and relaxation tissue doppler velocities (Figures 11 and 12), sometimes detectable even before development of left ventricular hypertrophy (LVH). Tissue Doppler Imaging (TDI) can provide a preclinical diagnosis of Fabry cardiomyopathy [89,90] and myocardial function can be quantified by ultrasonic strain rate imaging to assess radial and longitudinal myocardial deformation (Figures 11 and 12) [91].

##### Right ventricular structural changes

Right ventricular hypertrophy (RVH) with normal chamber size and preserved systolic but impaired diastolic function represents the typical right ventricular (RV) structural change in FD. When a detailed echocardiographic examination was performed in 58 patients with FD (mean age 40 ± 16 years), RVH was present in 40% of affected subjects with similar prevalence in both genders [92]. Two thirds of patients with LVH also exhibited RVH. RV dilatation was not present in any subject. RV diastolic dysfunction was present in 47% of 45 subjects in whom RV filling was assessed. RV diastolic dysfunction was associated with the presence of RVH. A significant correlation between RV wall thickness and age and left ventricular mass index was noted [92]. In another study, the degree of right ventricular involvement in FD was also related to the left ventricular cardiomyopathy stage [93]. RV involvement is common in FD and ultimately progresses to severe diastolic RV dysfunction. These findings might explain why patients with preserved left ventricule (LV) function can develop clinical features such as reduced exercise capacity, organomegaly and lymphoedema [94].

##### Electrocardiographic abnormalities

**Figure 13:**
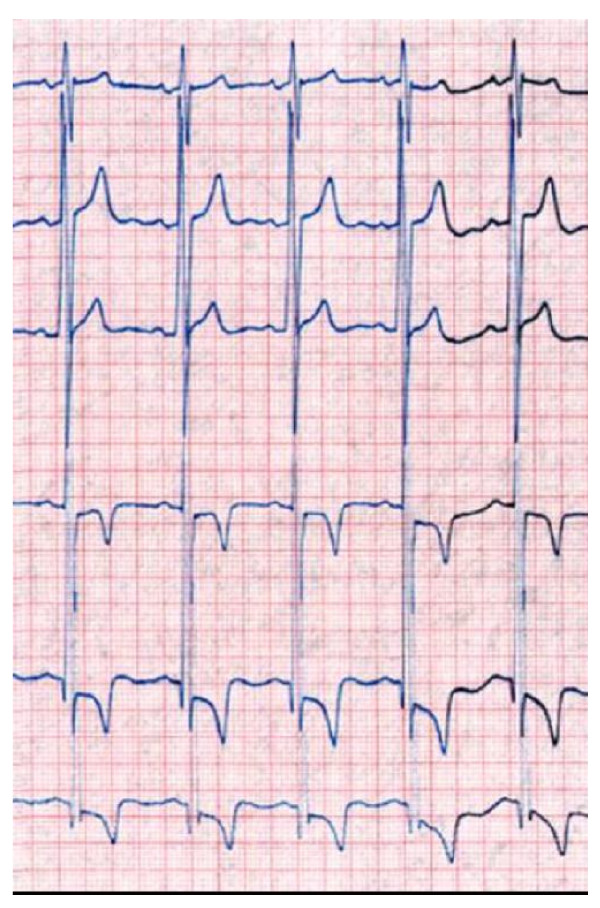
**ECG:** showing electrical signs of left ventricular hypertrophy with increased Sokolow index, depressed ST segment and negative T waves in left derivations

**Figure 14:**
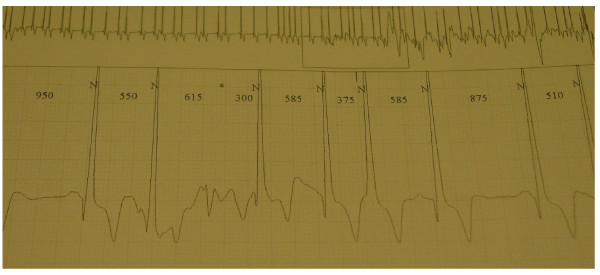
**24-hour-ECG holter**: is recommended at baseline and during follow-up of enzyme replacement therapy if arrhythmia is suspected on ECG or palpitations are reported by the patient.

Electrocardiographic (ECG) changes in patients with FD are frequent and include voltage criteria and repolarization changes related to LVH and/or remodeling, ST segment depression and T-wave inversions [95]. Other abnormalities include a short PR interval (< 0.12 msec) [96] due to a short P wave, enlarged QRS complex and prolonged QT_C _intervals, intermittent supraventricular tachycardia [97], AV node blocks [98], bundle branch blocks [99] and arrhythmias (Figure 13) [19,78-81]. 24-hour-ECG holter is therefore useful and recommended at baseline and during follow-up of enzyme replacement therapy (ERT) (Figure 14). The cardiac manifestations observed in patients with classic FD are also observed in patients with the cardiac variant of FD [28,100].

##### Valvular involvement

Although previous work reported a high prevalence of mitral valve prolapse in Fabry patients [101], this finding was not confirmed by recent studies [80,102].

##### Coronary involvement

The myocardial perfusion reserve was found to be significantly reduced in patients affected with FD [103]. Patients with FD have abnormal coronary microvascular function [82].

##### Exercise capacity

Exercise capacity is reduced in patients with FD compared with that predicted from normative population data [104,105].

##### Autonomic dysfunction

Fabry patients have autonomic dysfunction but usually do not present clinically overt signs of orthostatic dysregulation [106].

##### Aortic root dilatation

**Figure 15:**
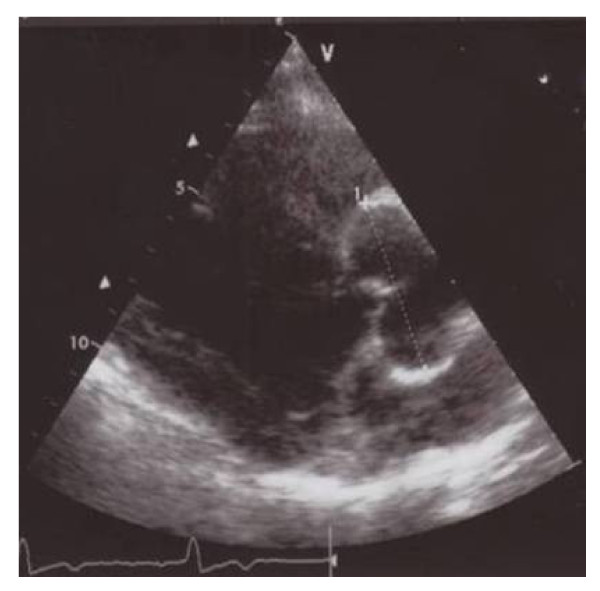
**Aortic root dilatation:** echocardiography shows aortic root diameter of 47 mm in a 51-year-old male patient with Fabry disease. Courtesy: Pr Olivier DUBOURG and Pr Dominique GERMAIN, University of Versailles - St Quentin en Yvelines (UVSQ), Versailles, France.

**Figure 16:**
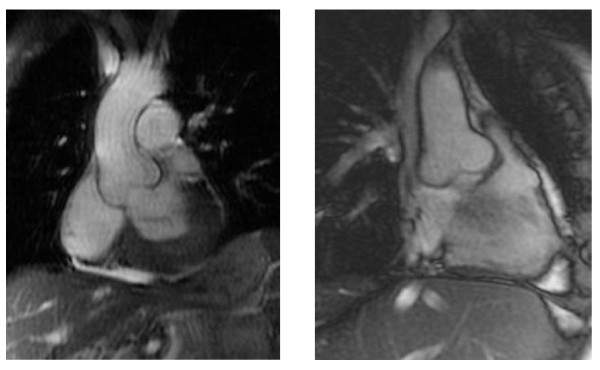
**Aortic root dilatation in a patient suffering from Fabry disease**: magnetic resonance imaging (MRI) showing aortic root dilatation in Fabry disease. Pr Dominique GERMAIN, University of Versailles - St Quentin en Yvelines (UVSQ), Versailles, France

FD is associated to an increased risk of developing aortic root dilatation in male patients [107]. Aortic root dilation was detected in 24% of 71 hemizygous male patients and was statistically associated with the presence of a dolicho-ectatic basilar artery (p = 0.008) (Germain DP, unpublished data) (Figures 15 and 16) [107].

#### D. Cerebrovascular lesions

**Figure 17:**
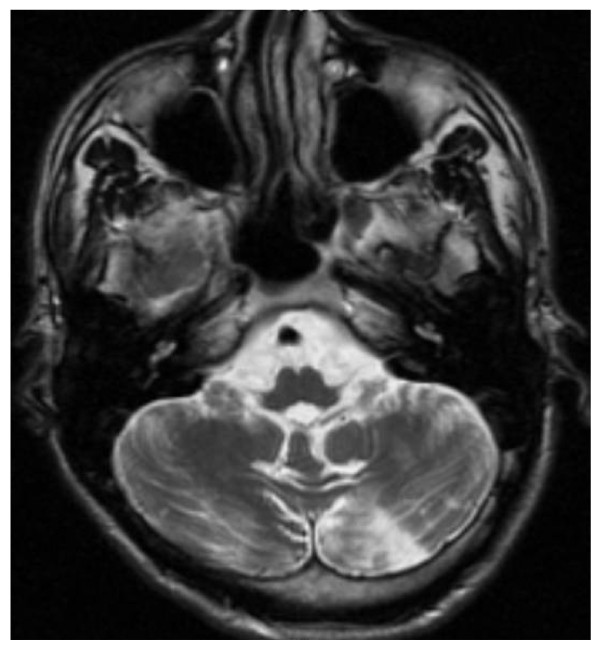
**Stroke in a patient affected with Fabry disease**: axial brain MRI section showing stroke of the left cerebellar hemisphere that revealed Fabry disease in an otherwise asymptomatic 27-year-old male patient.

**Figure 18:**
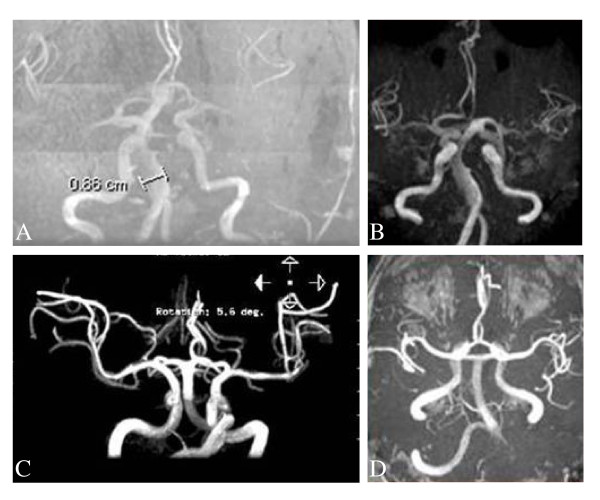
**Dolichoectasia of the vertebro-basilar circulation**: time of flight magnetic resonance angiographies showing ectatic vessels in four patients affected with Fabry disease.

The early peripheral neuropathic hallmarks of FD [38,108,109] are often followed by cerebrovascular complications and autonomic dysfunction in adulthood. Some of the most devastating neurological features of FD are caused by cerebrovascular lesions - the result of multifocal involvement of small blood vessels [110,111]. Cerebrovascular involvement can lead to a wide variety of signs and symptoms, ranging from mild to severe, including headache, vertigo/dizziness, transient ischemic attacks, ischemic strokes (Figure 17) [111-113] and more rarely vascular dementia [114,115]. Using data from the Fabry Registry^®^, the prevalence of strokes in FD was estimated to be 6.9% in males and 4.3% in females, much higher than in the general population. Median age at first stroke was 39 in men and 46 years in women and stroke may be the first manifestation of the disease [111]. There is a high prevalence of hypertension, cardiac disease and renal disease in patients who have had a stroke in the context of FD [111]. Data from both the Fabry Registry^® ^[111] and the Fabry Outcome Survey^® ^(FOS^®^) [110] have shown that the majority of strokes in FD are due to small vessel events. A dilative arteriopathy of the vertebrobasilar circulation has also been documented (Figure 18) [112,116]. Thrombus formation may be enhanced in FD due to the adhesion of neutrophils and monocytes to endothelial cell walls [117] or to changes in the regional cerebral hyperperfusion [118-120]. Serum myeloperoxidase level has been found to predict the risk of a vasculopathy-related event in males affected with FD [121].

**Figure 19:**
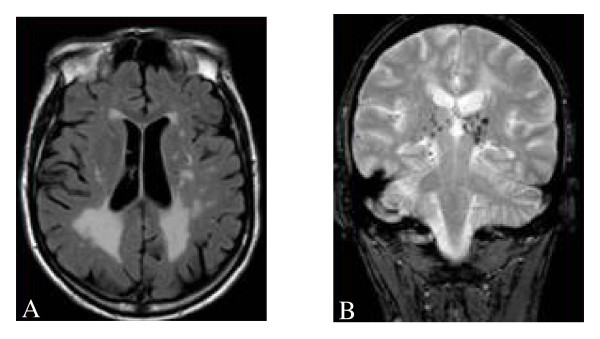
**Cerebral white matter hyperintensities, lacuna and microbleeds**: A. Fluid-attenuated inversion recovery (FLAIR)-weighted axial MRI section showing multiple white matter lesions in the cerebral hemispheres in a 53-year-old male patient who had a Fazekas score of 9. B. Lacuna and microbleeds in the same patient. Courtesy: Dr Robert CARLIER and Dr Frédéric COLAS, CHU Raymond Poincaré, Garches, France.

**Figure 20:**
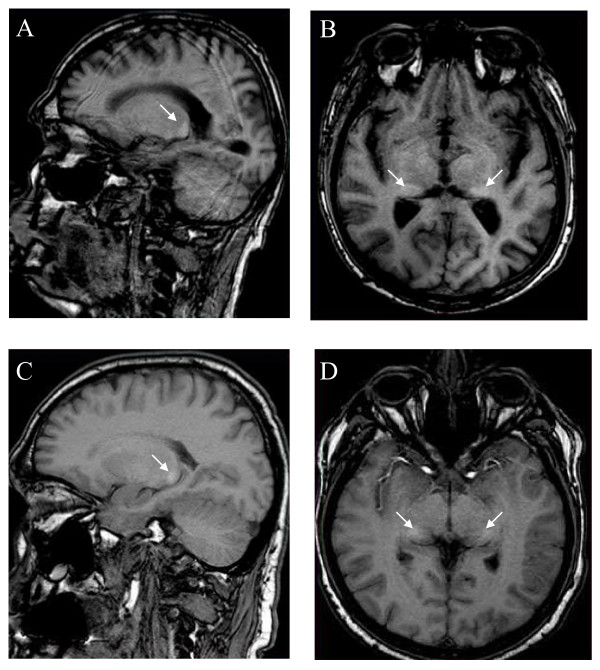
**The pulvinar sign**: T1-weighted sagital (A) and axial (B) MRI sections showing the pulvinar sign in a 66 year-old male patient. T1-weighted sagital (C) and axial (D) MRI section showing symmetrical high signals in the pulvinar region in a 42-year-old male patient. Courtesy: Dr Robert CARLIER and Dr Frédéric COLAS, CHU Raymond Poincaré, Garches, France.

Imaging modalities that can be used to explore cerebrovascular involvement in Fabry patients include MRI [116], trans-cranial Doppler (TCD) [122], proton MR spectroscopy (MRS), positron emission tomography (PET) and diffusion tensor imaging [123]. White matter lesions may be single, multiple or confluent on MRI (Figure 19) [124,125]. In addition, diffuse neuronal involvement, extending beyond the areas of MRI-visible cerebrovascular abnormalities has been found, and in such cases, 1H-MRS may be the preferred modality [126]. Cerebral MRI can reveal periventricular white-matter lesions, microbleeds (Figure 19), cortical grey-matter infarcts and deep lacunar infarcts in both grey and white matter [111,127-130]. Some patients affected with FD have an aseptic meningitis [113,131,132]. Hyperintensity in the pulvinar on T1-weighted images is a common finding in FD, likely reflecting the presence of calcification [133,134]. Recent findings suggest that the pulvinar sign is a highly specific sign, distinctively characteristic of FD [135], more frequent in male patients with cardiomyopathy and severe kidney involvement (Colas F, Carlier RY and Germain DP, unpublished data) (Figure 20).

In a pilot study, head MRI was performed in a cohort of 44 consecutive hemizygous male patients and 7 heterozygous females affected with FD. Chiari type I malformation was identified in 6 individuals (3 males and 3 females) [136]. Whether the association is coincidental or not, does need further studies but Chiari malformation may explain the episodes of headache frequently encountered in FD and should be ruled out in all Fabry patients [136].

Comprehensive neurological evaluation is essential before the institution of ERT, to assess disease extent and severity. Frequency and severity of pain should be assessed using tools such as the Brief Pain Inventory (BPI) or the McGill Pain Inventory. Clinical investigations include brain imaging by MRI with T1, T2 and FLAIR-weighted images and magnetic resonance angiography (MRA) may be indicated to exclude cerebral vasculopathy. Laboratory evaluation of comorbid stroke risk factors may identify patients with significantly elevated homocysteine, with a vitamin deficiency state, or with other genetic prothrombotic risk factors [51].

#### E. Auditory and vestibular abnormalities

**Figure 21:**
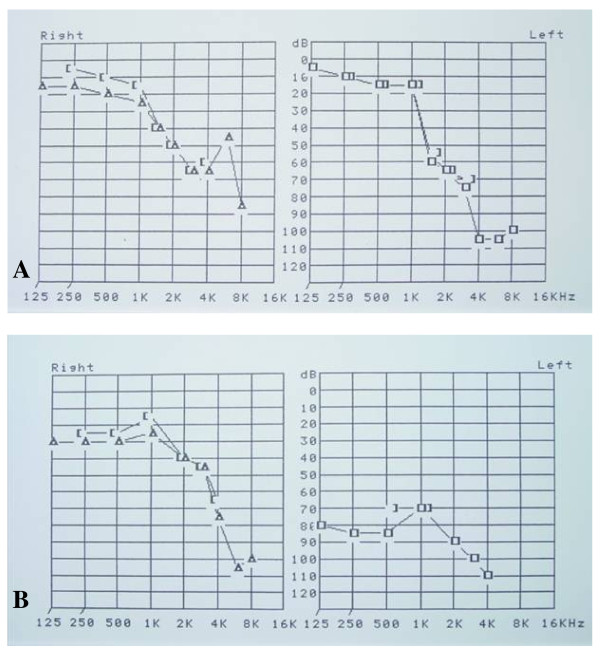
**Hypoacousia in patients affected with Fabry disease**: A. Hypoacousia in a 39-year-old male with hypertrophic cardiomyopathy, cerebral lacuna and kidney transplant. B. Sudden deafness of the left ear and bilateral hypoacousia in a 54-year-old male patient with tinnitus, vertigo, vertebro-basilar TIA, hypertrophic cardiomyopathy and kidney transplant. Courtesy: Dr Philippe AUBERT and Dr Karelle BENISTAN, CHU Raymond Poincaré, Garches, France.

Auditory and vestibular abnormalities are frequent deficits observed in FD, resulting in a range of symptoms, such as hearing loss [137,138], tinnitus and vertigo [137,139]. The high incidence of both progressive hearing loss and sudden deafness in male patients affected with classic FD has been demonstrated (Figure 21) [137]. A correlation of neuropathic and vascular damage with hearing loss was found in males in whom residual **α**-galactosidase A activity appears to have a protective effect against hearing loss [140]. Progressive vestibular loss was found in 80% of males and 77% of females when assessed with head impulse testing [141].

#### F. Ocular manifestations

**Figure 22:**
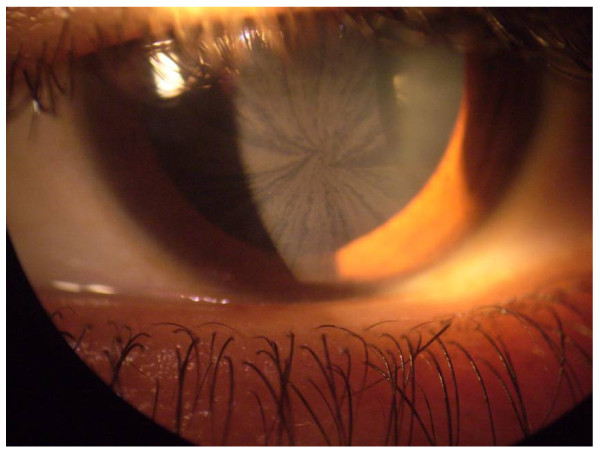
**Cornea of a female patient heterozygote for FD**: sub-epithelial brown lines show the typical pattern of so-called "*cornea verticillata*". These opacities do not impair the visual acuity. Courtesy: Dr Juan-Manuel POLITEI, Buenos-Aires, Argentina.

**Figure 23:**
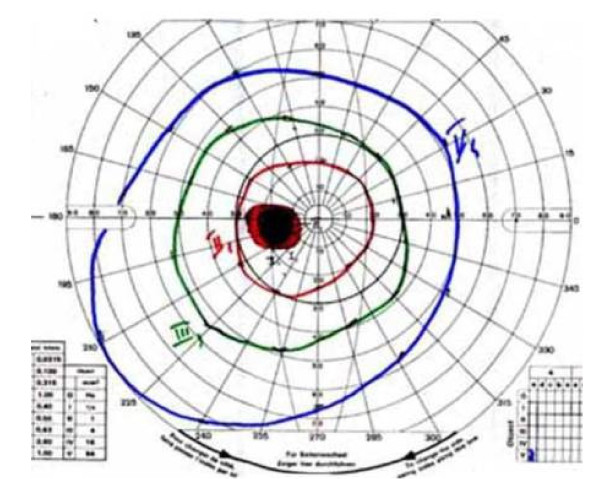
**Standard Goldman visual field of the left eye of a patient affected with Fabry disease**: the blind spot is enlarged. Courtesy: Dr Christophe ORSSAUD, Paris, France.

Corneal opacities (visible by slit-lamp microscopy) are the most common and early of ocular signs, occurring in almost all hemizygous males (Figure 22) [142-144]. It should be noted, however, that treatment with amiodarone or chloroquine can produce similar ophthalmological signs [145]. Mild to marked tortuosity of the conjunctival and retinal vessels is also observed in patients with FD [142,143]. Neither corneal dystrophy nor retinal/conjunctival lesions impair visual acuity; however, acute visual loss caused by unilateral occlusion of the central retinal artery has been reported [146]. Anterior and posterior subcapsular cataracts are also observed, the latter also being termed the 'Fabry cataract' in that it represents a pathognomonic ocular sign of FD. More recently, an enlargement of the blind spot (Figure 23) was reported in 38.7% (n = 27) of patients, although this was not associated with any defects in colour vision [142].

#### G. Respiratory involvement

Respiratory involvement, manifesting as dyspnea with exercise, chronic cough and wheezing, is frequent in both genders with FD [147,148]. A recent study has found the prevalence of airway obstruction in FD to be 26% in women and 61% in men [149,150]. A clinically relevant age- and gender-dependent progressive pulmonary involvement in FD patients has been demonstrated [150] and the effects of ERT on pulmonary involvement are currently being investigated. Recently, ERT was shown to stabilize obstructive pulmonary FD associated with respiratory Gb_3 _storage in one heterozygous female [151].

In another study, 39 patients with a diagnosis of FD underwent pulmonary function testing (spirometry), and a non-invasive cardiopulmonary exercise test. A control group was selected for comparison. Eighteen of the 39 Fabry patients (46%) exhibited a significant decrease in diastolic blood pressure (DBP) during exercise. The drop in DBP was evident in 9 of the 24 female patients (38%). None of the control patients had a significant drop in DBP during exercise. The finding of a significant decrease in DBP in patients with FD may explain deficits in exercise tolerance [104].

#### H. Skeletal involvement

**Figure 24:**
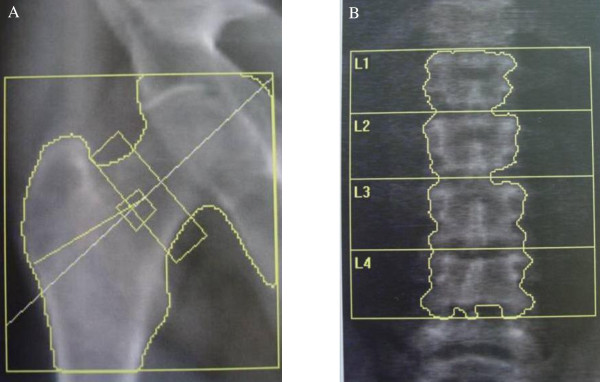
**Dual-energy X-ray absorptiometry (DEXA) assessment of bone mineral density of the femoral neck (A) and the lumbar spine (B)**: T scores of - 4.2 and - 4.3 were found at the hip (A) and lumbar spine (B), respectively in a 53 year-old male patient affected with Fabry disease. Courtesy: Dr Caroline LEBRETON, CHU Raymond Poincaré, Garches, France.

**Figure 25:**
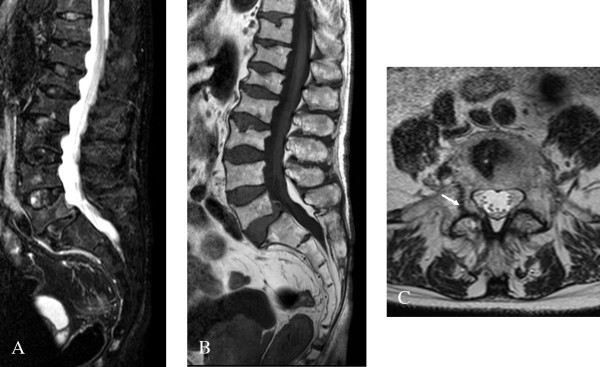
**Bone magnetic resonance imaging in a Fabry patient with severe osteoporosis**: A (STIR, sagittal view) and B (T1, sagital median): several vertebral body fractures are seen, without signal anomaly in T1 or T2 in favor of ancient fractures. A mild spondylolisthesis of L5 on S1 can be observed. C (T2, axial view): fracture of the right pedicula of L5 (arrow) in a 72-year-old patient with severe osteoporosis. Courtesy: Dr Robert CARLIER, CHU Raymond Poincaré, Garches, France.

In a recent study, bone mineral density of the lumbar spine and the femoral neck was assessed by dual-energy X-ray absorptiometry (DEXA) in 23 hemizygous male patients with a mean age of 31 years (range: 16-60 years) affected with classic FD. Using the World Health Organization classification, 20 of the 23 patients (88%) with FD had either osteopenia (n =11) or osteoporosis (n = 9) at one or both sites (Figure 24) [152,153]. Skeletal involvement has been subsequently confirmed in a larger cohort of 53 patients in which osteopenia was present in approximately 50% of cases [154]. Cases of severe osteoporosis with spontaneous lumbar fractures have recently been described (Figure 25) [155]. Patients suffering from Fabry disease should follow current recommendations regarding identification and treatment of vitamin D deficiency.

#### I. Depression and quality of life

Depression is a frequent and under-reported problem in patients with FD [156]. As many as 46% and 28% of patients may have depression and severe clinical depression, respectively [47]. Most patients identified in recent surveys were undiagnosed for depression, which underscores the need for the correct assessment of depressive symptoms in patients with FD. Since this is an under-recognized problem, the benefits of treatment are unknown. Depression can seriously impact quality of life in patients with FD. Reduction in quality of life has been demonstrated using a variety of questionnaires including the SF-36, EuroQoL and MMPI-2 [46,157-159]. Psychiatric and neuro-psychological evaluations have been recommended in the assessment of patients with FD [160,161].

#### J. Miscellaneous

##### Anemia

Data from the FOS^® ^and the Fabry Registry^® ^show that mild peripheral blood cytopenias, particularly anemia [162], are prevalent among patients with FD [163].

##### Arterial remodeling and intima-media thickening

Large artery phenotype (arterial wall structure and function) was non-invasively investigated in 21 hemizygous patients with FD and 24 age-matched male controls. Common carotid and radial artery diameter, intima-media thickness (IMT) and distensibility were determined with high-definition echotracking systems and aplanation tonometry. Patients with FD had a significant twofold increase in radial artery IMT and distensibility, independent of body surface area, age and mean blood pressure. Radial artery IMT increased significantly with age in each group. However, the slope was 2.3-fold higher in FD patients than in controls (p < 0.001). Common carotid artery (CCA) IMT was mildly but significantly increased in patients with FD (+18%), whereas distensibility was unchanged [164,165].

Another study presented evidence of a major increase in CCA IMT, both in hemizygous and heterozygous patients with FD, in the absence of focal atherosclerotic plaques [166]. The authors examined the possible correlation between left ventricular hypertrophy and IMT of the common carotid artery. Thirty male and 38 female patients were enrolled. LVH was found in 60% of men and 39% of women. Increased CCA IMT was equally present in males and females. LVH and CCA IMT occurred concomitantly in FD suggesting common pathogenesis. The underlying cause may be a circulating growth-promoting factor whose presence has been confirmed *in vitro *[167].

##### Azoospermia

Testicular biopsies performed in two infertile men suffering from FD with azoospermia revealed characteristic aspects of trihexoside ceramide (Gb_3_) deposits in Leydig cells by optical and electronic microscopic analysis [168].

##### Facial dysmorphism

Although facial dysmorphism is not a prominent sign in FD, minor facial abnormalities have been previously reported. By analysing three-dimensional images of faces, facial dysmorphology was quantified in a cohort of both males and females affected with FD. Morphometric analysis of different regions of the face revealed significant differences in face shape in male patients and to a lesser extent in female patients. In male patients, the most prominent abnormalities were located in the peri-orbital region. Pattern recognition techniques achieved a discrimination accuracy of up to 85% for male patients compared with healthy controls. The discrimination accuracy in female patients only reached 67% [169].

##### Hypothyroidism

In a small study, subclinical hypothyroidism (normal serum free thyroxine concentrations along with elevated serum TSH levels) was found in 4 of 11 patients (36.4%) who were investigated [170]. An endocrine work-up should be recommended in all patients suffering from FD [171].

##### Lymphoedema

Lymphoedema, already mentionned in one of the original papers on FD [1], has since been observed in a number of patients [58] and linked to structural and functional changes of the lymphatic microvessels of the skin [172].

##### Parapelvic kidney cysts

Twenty-four patients who were enrolled in an enzyme replacement trial underwent prospective renal imaging evaluation with kidney MRI and computed tomography (CT). Nineteen age-matched healthy controls were concurrently enrolled in this cross-sectionnal, case-control study. The presence and localization of kidney cysts as well as the ratio of the signal intensity between medulla and cortex were determined. Fifty percent of FD patients had renal sinus cysts, compared to one individual (7%) in the control group. The cause of such cysts in FD remains to be elaborated [173] but they may contribute to earlier recognition of the disease [174].

##### Priapism

Cases of priapism have been observed in young boys affected with FD. Conventional treatment with cavernovenous shunting was only partly successful in one case, and percutaneous gelfoam embolization of the internal pudendal artery may prove a better option [175]. Additional cases of priapism associated with FD were identified through a search of the literature [176].

#### K. Heterozygous females

Traditionally, it was considered that heterozygotes did not develop symptoms and heterozygous females were erroneously described as "carriers of the defective gene" who were more or less safeguarded against developing disease symptoms. However, an increasing number of publications and evolving knowledge about the natural course of disease indicate that the term X-linked recessive should probably be discontinued and FD simply described as following "X-linked inheritance" [177,178].

Clinical signs and symptoms vary widely in heterozygous females. This phenotypic heterogeneity is thought to be partly due to lyonization [179], a process whereby one copy of the X-chromosome is randomly inactivated in all cells of the female embryo, so that heterozygous females are essentially a 'mosaic' of normal and mutant cells in varying proportions. In X-linked diseases, heterozygous females may be symptomatic, probably as a consequence of skewed X-chromosome inactivation, which results in a higher percentage of the × chromosome bearing the mutant gene being expressed in the particular tissue of importance. Such variability in symptom severity is characteristic of X-linked heterozygotes [180] and should be kept in mind when assessing and diagnosing potential patients.

The clinical spectrum in females ranges from a seemingly asymptomatic disease course occasionally observed to the "classic" severe phenotype observed in males, with a variety of clinical presentations in between [24,26,181-183]. Heterozygotes may display all symptoms of the disease including pain [184], orthostatic hypotension [185], angiokeratoma [53], ocular abnormalities [186], cochleovestibular involvement [51,139], gastrointestinal symptoms [50] and respiratory involvement [150]. A high percentage of females develop vital organ damage involving the heart [26,78,79,96,187,188], brain [111,129,189-191] and, more rarely, kidneys [26,32,73,76,186] about a decade later than males [24,184]. Of the 1077 enrolled females in the Fabry Registry^®^, 69.4% had symptoms and signs of FD. The median age at symptom onset among females was 13 years, and twenty percent experienced major cerebrovascular, cardiac, or renal events, at a median age of 46 years [24].

In a retrospective chart review of 279 affected males and 168 females suffering from Fabry disease, the mean rate of estimated glomerular filtration rate (eGFR) decline for patients was -1.02 ml/min/1.73 m^2^/year for females as compared to -2.93 ml/min/1.73 m^2^/year for males and advanced Fabry nephropathy was less prevalent and occurred later among females than males [25].

Altogether, females with FD have a significant risk for major organ involvement and decreased quality of life [158], and should be regularly monitored for signs and symptoms of FD [24,51].

#### L. Atypical variants

FD has long been regarded as a *full-blown *multisystemic disease with most, if not all, affected males developing a "classic" phenotype. Later on, the sub-classifications "cardiac variant" [29] and "renal variant" [30] were introduced for patients with predominant cardiac or renal involvement, respectively. In high-risk adult populations, screening efforts have been shown to be effective in diagnosing Fabry patients among individuals with end-stage renal disease [30,192,193], unexplained cardiac hypertrophy [194-196] or strokes in young people with no apparent predisposing factors [197-200]. Screening of patients with atherosclerosis [201] or ophthalmological screening [202] may be of less value.

Atypical variants have few or none of the hallmark symptoms of classical FD, but have manifestations confined predominantly to one organ system [28,100]. Presenting much later in life (fourth to sixth decades) than patients with classical disease, they are often identified serendipitously. In contrast to their classically affected counterparts, atypical variants have residual **α**-galactosidase A activity that varies between 2 and 20% of normal [35,203,204].

##### Cardiac variant

The cardiac variant - the most widely reported atypical variant - presents with cardiac manifestations in the absence of overt systemic involvement [28,29,100]. Manifestations include cardiomegaly, electrocardiographic abnormalities consistent with cardiomyopathy, non-obstructive hypertrophic cardiomyopathy and myocardial infarctions; mild proteinuria may also be detected.

The cardiac variant was initially thought to be rare, but a Japanese study of 1603 males undergoing routine echocardiography found that 7 (3%) of 230 patients with left ventricular hypertrophy had clinically unsuspected FD [29]. Furthermore, recent reports suggested that FD should also be considered in all cases of unexplained homogeneous hypertrophic cardiomyopathy [194-196]. In a British study, 6 of 153 males (4%) consecutively referred with hypertrophic cardiomyopathy were found to have **α**-galactosidase A levels diagnostic of FD [194]. In a Spanish study, 0.9% of males and 1.1% of females with hypertrophic cardiomyopathy were diagnosed with FD [195].

##### Renal variant

There are also reports of hemizygous males with disease manifestations confined to the kidney. Renal variants have been identified among Japanese chronic dialysis patients whose end-stage renal disease had been misdiagnosed as chronic glomerulonephritis [30]. The patients had absent or low **α**-galactosidase A activity, and were, subsequently, found to have *GLA *gene mutations [30]. These findings suggest that cases of FD may be underdiagnosed among renal dialysis [192] and transplant [205] patients. Their early detection is important since these patients may later develop vascular disease of the heart or brain. However, a much lower prevalence of FD (0.22%) was found in both a Dutch [206] and another Japanese [193] study performed in similar high-risk groups of hemodialyzed patients.

##### Intermediate variant

Presentation and clinical course can vary within the aforementioned phenotypes, and an intermediate phenotype has been described in which patients, in the absence of cardinal signs of FD in childhood, presented with a cardiac variant with hypertrophic cardiomyopathy and arrhythmia around age 40 but subsequently progressed to end-stage kidney failure [207].

### V - Etiology

#### A. Genetics

FD is transmitted as an X-linked trait. Contrary to the misconception that females will be marginally affected given the X-chromosome linked inheritance pattern, many heterozygotes will develop early symptoms and, later on, vital organ involvement [24,26,182]. The use of the term X-linked 'recessive' is therefore misleading and should be discontinued and FD described as following X-linked inheritance [177,208].

#### B. Gene location

Lysosomal **α**-galactosidase A (EC 3.2.1.22) is coded by a unique gene, *GLA*, whose locus is situated on the long arm of chromosome X, in position Xq22. The *GLA *gene consists of seven exons distributed over 12,436 base pairs (bp). There is extensive allelic heterogeneity, but no genetic locus heterogeneity.

#### C. Molecular pathology

**Figure 26:**
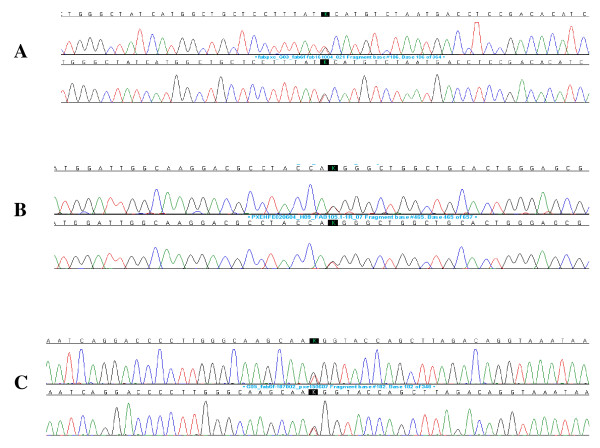
**Genotyping of the *GLA *gene in heterozygous females**: A. Patient CB, a 17-year-old girl, was shown to carry a T to G transversion in exon 6 at position 884 in the cDNA sequence. This nucleotide substitution alters the codon (TTC) for phenylalanine to the codon (TGC) for cysteine at position 295 of the **α**-galactosidase A protein (p.Phe295Cys). B. Patient ZB, a 46-year-old woman, was shown to carry a T to G transversion in exon 1 at position 125 in the cDNA sequence. This nucleotide substitution alters the codon (ATG) for methionine to the codon (AGG) for arginine at position 42 of the **α**-galactosidase A protein (p.Met42Arg). C. Patient NL, a 63-year-old woman was shown to carry a G to T transversion in exon 6 at position 982 in the cDNA sequence. This nucleotide substitution alters the codon (GGG) for glycine to the codon (TGG) for tryptophan at position 328 of the **α**-galactosidase A protein (p.Gly328Trp). Despite scanning of the rest of the gene, no other sequence abnormality was found. Courtesy: Pr Xavier JEUNEMAITRE and Dr Anne-Laure FAURET, HEGP, Paris, France.

FD can be caused by a variety of missense or nonsense point mutations, splicing mutations, small deletions or insertions [203,204,209-236], and large deletions [237,238]. The defects in the *GLA *gene encoding **α**-galactosidase A are heterogeneous with over 585 mutations recorded [239,240]; the majority of these mutations render the enzyme non-functional [239]. Most families have unique mutations potentially explaining the marked variability in the residual enzyme activity but only in part the natural course of the disease since intra-familial variability does exist. Novel **α**-galactosidase A mutations have been recently identified by our research group [e.g. p.Met42Arg (c.125T > G) (Figure 26), p.Gly43Ser (c.127G > A), p.Gly132Glu (c.395G > A), p.Lys168Asn (c.504A > C), p.Gln212Stop (c.634C > T), p.Phe295Cys (c.884T > G) (Figure 26), p.Leu300Pro (c.899T > C), and p.Gly328Glu (c.983G > A), D.P. Germain, unpublished data]. Non pathological single nucleotide polymorphisms such as c.-30G > A, c.-12G > A, and c.-10C > T in the 5' untranslated region (5'UTR), p.Asp313Tyr in exon 6 [241] and other sequence variations (VNTR) have been described [239,242,243]. Whether some published sequence changes, such as p.Arg112His, are true mutations or polymorphisms is still a matter of debate [244].

#### D. Structure of human α-Galactosidase A

The three-dimensional structure of human **α**-galactosidase A was determined by x-ray crystallography. The crystal structure showed a homodimeric molecule with each monomer containing two domains. The N-terminal domain is a classic (**β**/**α**)_8 _barrel, and the C-terminal domain contains eight antiparallel **β **strands packed into a **β **sandwich. Residues 32-328 comprise the N-terminal domain, and residues 329-421 fold into the C-terminal antiparallel domain. The N-terminal domain contains the active site, which is located at the C-terminal end of **β **strands **β**1-**β**7, near the center of the **β **barrel. Three N-linked carbohydrates are found on the surface of the molecule, away from the location of the active site and away from the dimer interface. The carbohydrate residues attach to aspartic acid residues N139, N192 and N215 and extend from the surface of the molecule [245]. The enzyme folds into a three dimensional fold that gathers 15 residues into an active site configuration specific for **α**-galactosides. The active site is formed from side chain residues of W47, D92, D93, Y134, C142, K168, D170, C172, E203, L206, Y207, R227, D231, D266, and M267. Residues C142 and C172 make a disulfide bond. The two active sites in the dimer are separated by approximately 50 Å [245]. The **α**-galactosidase A enzyme uses a double displacement reaction mechanism, where two consecutive nucleophilic attacks on the anomeric carbon of the substrate lead to breakage of the glycosidic linkage with overall retention of the anomer of the product. In human **α**-galactosidase A, the catalytic nucleophile is D170 and the catalytic acid/base is D231 [246].

### VI - Diagnosis

Early onset of FD signs and symptoms warrant prompt diagnosis, particularly because ERT is available. However, recognizing the early manifestations in clinical practice may be challenging due to a variety of reasons. The disease presentation is generally heterogeneous, symptoms may resemble more common diseases, and major renal or cardiac dysfunction is uncommon in pediatric patients. Nowadays, diagnostic delays may still be considerable and patients often have to visit several medical specialists before a correct diagnosis is made. Recent data showed that the overall diagnostic delays were ~15 years for both genders [24]. If clinical examination raises a suspicion of FD, appropriate biochemical and/or genetic confirmation is needed [247].

#### A. Biochemical diagnosis

##### Enzymatic assay

The demonstration of a deficient activity of **α**-galactosidase activity in plasma or leukocytes is the reference laboratory method which should systematically be used to confirm the clinical diagnosis of FD in males in whom the result will be conclusive [248]. Plasma assay may occasionally lead to false diagnosis and should be confirmed by a leukocyte assay [249]. In contrast, affected girls and adult females may have their enzyme activity falling within the normal range [250]. Therefore, all females should have their status determined by genotyping (analysis of the *GLA *gene mutation) [208].

A fluorimetric method that uses filter paper cards containing dried blood spots instead of the leukocyte pellet as the enzyme source was recently introduced for enzymatic diagnosis, allowing storage of the samples for up to 6 months due to stability of the enzyme [251-255].

##### Globotriaosylceramide measurement

Plasma Gb_3 _has also been proposed and used in the biochemical diagnosis of FD, but this method is time-consuming and, in females, plasma Gb_3 _levels are generally lower than in males and usually in the normal range [256].

Urinary Gb_3 _is a more reliable marker allowing diagnosis in the majority of both male and female patients [257-260]. However urinary Gb_3 _is not elevated in some patients with late-onset variants and/or particular mutations in the *GLA *gene (p.Asn215Ser) [261-263].

The analysis of tissue glycolipid composition [264] and the use of atmospheric pressure photoionization mass spectrometry (APPI-MS) for the analysis of Gb_3 _molecular species [265] and MALDI-TOF imaging of biomarkers [266] are not done routinely and are confined to research laboratories.

#### B. Genotyping

**Figure 27:**
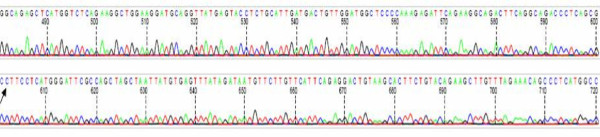
**Sequencing of PCR products obtained from amplification of DNA directly eluted from a 3-mm punch of dried blood spot (DBS) on filter paper**: a 60-year-old man with left ventricular hypertrophy of unknown origin was enrolled in a screening protocol for FD. Markedly decreased **α**-galactosidase activity was found on DBS. Using a second DBS, the patient was subsequently shown to carry a T to C transition in exon 2 at position 337 in the cDNA sequence of the *GLA *gene (c.337T > C). This nucleotide substitution alters the codon (TTT) for phenylalanine to the codon (CTT) for leucine at position 113 of the **α**-galactosidase A protein (p.Phe113Leu). Pr Dominique GERMAIN, University of Versailles - St Quentin en Yvelines (UVSQ), Versailles, France

In female heterozygotes, a-galactosidase activity may be within the normal range [250,252] and therefore, the definitive diagnostic confirmation should be made by genetic analysis in suspected cases (Figure 26). The publication of the complementary (cDNA) [267] and genomic DNA [268] sequences of the *GLA *gene (Genbank X14448) has paved the way towards understanding of the molecular basis of FD. Direct molecular analysis is easy because of the small size of the gene and allows the precise characterization of the mutation of the *GLA *gene. A method that uses filter paper cards containing dried blood spots instead of the leukocytes pellet as the source of DNA was recently developed for sequencing, allowing genotyping from a dried blood spot on filter paper to confirm enzymatic diagnosis (Figure 27) [196].

Denaturing high-performance liquid chromatography (DHPLC) has been shown to be useful as a screening method [269]. Since direct sequencing limited to exons may miss deletions, the use of Multiplex Ligation-dependent Probe Amplification (MLPA) has been recommended in cases where a decreased enzyme activity is not associated with the identification of a pathogenic point mutation [270].

#### C. Screening

Screening individuals with a family history of FD or newborn screening programs are the only practical ways of identifying patients before the development of symptoms. Moreover, screening of patients in high risk groups who may be exhibiting late-onset symptoms of FD but who have not been diagnosed may be key in optimizing the management of disease in these patients.

Any screening requires a reliable and preferably rapid and low-cost method. Measurement of the accumulated urinary Gb_3 _has been proposed [258], but its reliability as a biomarker of FD, particularly in females, is unproven [262,271]. Screening of at-risk groups is often conducted by measuring plasma **a**-galactosidase A activity, but clinicians should be aware that this can fail to detect all cases of FD [272]. Identification of the deficient enzyme activity in dried blood spots (DBS) may be a more reliable method of screening for FD and this approach has been validated in males [198,250-252,273,274] but fails to detect about one third of heterozygous females [250,252,253].

#### D. Histology

##### Light microscopy

The observation of biopsies with light microscopy does not usually contribute a great deal to diagnosis but lipid staining of kidney biopsies can reveal storage cells within glomeruli and, when electron microscopy (EM) is not being done or not available, semi-thin sections stained with toluidine blue or Masson's trichrome can allow diagnosis (Figures 3 and 4). However, given the number of false negatives and the non specificity of the results, this invasive procedure should not be used for diagnostic purpose.

##### Electron microscopy

Ultrastructural studies of endomyocardial and kidney biopsies can reveal lysosomal storage in cardiomyocytes or in a variety of kidney cellular types, respectively. The ultrastructural appearance of the inclusions is of whorled layers of alternating dense and pale material ('zebra bodies' or myelin figures) (Figures 5, 6 and 7). However, due to the invasive nature of the procedure and the availability of reliable biochemical or molecular methods, these procedures should be considered only in the rare instances where there is residual **α**-galactosidase A activity in males or doubts on the causality of a DNA sequence change in females. Skin biopsy observed by EM may be a useful additional diagnostic test when carefully interpreted by an expert pathologist [275]. However, acquired metabolic disorders, such as the one induced by chloroquine therapy, may result in storage of ultrastructurally similar inclusions in many of the same cells as FD, leading to erroneous interpretation [276]. In addition, skin biopsies are often normal in heterozygous females and therefore not of great utility.

#### E. Ancillary markers

Although laboratory tests are usually normal, anemia [162], hyperhomocysteinemia [277], raised HDL cholesterol [278] and elevated Lp(a) (Germain DP, unpublished data) have been reported in a number of patients with FD. Urinary sediment examination can reveal casts, erythrocytes and cells containing accumulated Gb_3_. Elevated serum levels of B natriuretic peptide (BNP) and troponin IC have been found in patients with advanced left ventricular hypertrophy (Germain DP, unpublished data). 25(OH) vitamin D levels should be investigated in all patients suffering from FD since vitamin D deficiency is found in about 40% of them in France (Germain DP, unpublished data).

#### F. Biomarkers

One of the most urgent research needs is for (a) reliable and validated biomarker(s) with which to assess disease progression and treatment response. Ideally, measurement of such (a) surrogate marker(s) would involve non-invasive testing. Although various imaging techniques have shown promising results, the clinical relevance of what they reveal in patients with FD has yet to be evaluated for its correlation with clinical endpoints. There is currently no proper plasma or urinary biomarker for FD.

Mildly elevated plasma chitotriosidase levels have been reported in male patients but not in heterozygous females [279].

Globotriaosylsphingosine or lyso-Gb_3 _has been reported to be elevated in FD patients. This analyte is elevated in the plasma of hemizygous males and to a lesser extent in that of adult females with classical FD and lyso-Gb_3 _appears interesting to monitor enzyme replacement therapy [244,280]. Lyso-Gb_3 _was shown to be an independent risk factor for the development of cerebrovascular white matter lesions in male patients with FD while, in females, plasma lyso-Gb3 concentration correlated with overall disease severity [281].

Lyso-Gb_3 _could be a potential biomarker since plasma lyso-Gb_3 _level in Fabry patients who had received ERT was shown to be elevated at baseline and to fall more dramatically on ERT than that of Gb_3 _[282]. Urinary lyso-Gb_3 _may also prove a potential biomarker [283]. Lyso-Gb_3 _may have a role in glomerular injury in FD by promoting the release of secondary mediators of glomerular injury (Transforming growth factor-beta1 (TGF-**β**1) and the macrophage inhibitory factor receptor CD74) common to diabetic nephropathy [284].

Sphingosine-1-phosphate (S1P) was recently identified as a biologically active growth-promoting factor involved in cardiovascular remodelling in both males and females with FD [285]. Male patients had significantly higher plasma S1P levels compared with healthy controls. Moreover, there was a strong correlation between plasma S1P levels and LVM index, and increased common carotide artery IMT in patients with FD [285]. Sphingosine-1 phosphate has been shown to induce *in vitro *vascular smooth muscle cells proliferation by a variety of signal transduction pathways [285].

In the interest of future research, biobanking of plasma, serum and urine samples remains highly recommended in all patients affected with FD prior to initiation of ERT.

### VII - Differential diagnosis

In childhood, other possible causes of pain such as rheumatoid arthritis [286], rheumatic fever, systemic lupus erythematosus, Raynaud's disease, and 'growing pains' (a frequent misdiagnosis in children with FD) must be ruled out. In adulthood, celiac disease and multiple sclerosis [287] are the most often-cited differential diagnoses particularly in females. Similarly, when no mutation of the *GLA *gene has been identified, the possiblity of a phenocopy mimicking FD, should be considered [288].

Finally, whether a combination of several single nucleotide polymorphisms (SNPs) in the *GLA *gene leading to decreased but residual **α**-galactosidase activity may be a risk factor and predispose to hypertrophic cardiomyopathy and/or ischemic stroke, when combined with additional environmental or genetic factors, is unknown and warrants further studies.

### VIII - Genetic counseling

In contrast to the vast majority of lysosomal storage disorders, which are inherited in an autosomal recessive manner, FD, together with mucopolysaccharidosis type II (Hunter syndrome) and Danon disease (LAMP2 deficiency), is inherited as an X-linked trait [208]. Consequently, there is no male-to-male transmission of FD, but affected fathers will pass the defective gene to all their daughters, while heterozygous females have a 50% risk with each conception of transmitting the gene; sons who inherit the mutant gene from their mother will have the disease, while daughters will be heterozygotes who may or may not develop disease manifestations.

Once the diagnosis has been confirmed, the opinion of a geneticist should be sought and family screening carried out [289]. Pedigree analysis and effective screening of the family of a diagnosed (adult) patient is likely to result in identification of several previously unrecognized affected family members, including young relatives at a relatively early stage of their disease [208,290]. This provides the opportunity to offer genetic counseling and timely therapeutic intervention [290]. Appropriate family support should be provided which may be achieved through the help of patients' associations (Appendix).

### IX - Prenatal diagnosis

Biochemical or molecular prenatal diagnosis of FD is technically feasible by determination of **α**-gal A activity in direct and/or cultured chorionic villi at 10 weeks of pregnancy or in cultured amniotic cells at about 14 weeks of pregnancy, respectively. Determination of fetal sex using maternal blood at 9-11 weeks of pregnancy is occasionnaly used. Genetic counseling prior to prenatal diagnosis should be provided to discuss the options and risks since intra-familial phenotype variations, existence of atypical late-onset variants and recent availability of a specific therapy have singularly complicated genetic counseling and prenatal diagnosis. For ethical reasons, prenatal diagnosis of FD has always been controversial for female fetuses and has now become questionable even for males fetuses since the advent of ERT. There is limited experience with preimplantation diagnosis of FD, but the diagnosis has been performed successfully (no reports in the literature) [291].

### X - Management

FD is a paradigm of a multi-system condition and symptoms express themselves in many organs [25,51,292,293]. Maximal, comprehensive therapy for FD includes ERT [294-298], conventional medical treatment [51] and adjunctive therapies [181,299,300].

#### A. Conventional medical treatment and adjunctive therapies for Fabry disease related morbidities

Supportive care is important. The effective management of FD requires a multidisciplinary approach [301]. Symptom management in patients may consist of lifestyle modifications and prophylactic medications [51,299].

##### Pain

Patients with neuropathic pain may benefit from avoidance of circumstances triggering acute pain attacks, e.g. significant physical exertion and temperature changes. The neuropathic pain associated with FD can be managed with analgesics, but nonsteroidal anti-inflammatory drugs are generally ineffective (and potentially harmful for kidney function) while narcotic analgesics should be avoided [292] although this has been debated [302]. Carbamazepine [303,304], oxcarbazepin, gabapentin [299,305], pregabalin and phenytoin [306] are classically used to manage pain in FD (Table 3) [51,299]. Some patients use illicit drugs, particularly marijuana for pain control and GI manifestations, especially if their symptoms have been overlooked by doctors.

**Table 3:** Guidelines for baseline examination and follow-up of patients affected with Fabry disease

Organ/system	Assessment	Guidelines
**General**	General status, quality of life (SF36^® ^Health survey, EuroQOL or PedsQL^® ^measurement mode), school or work performance, depression, anxiety, drug use, somatic growth	Baseline (at first visit), every 12 months
		
	Complete physical examination	Baseline, every 12 months
		
	Genetic counseling	Baseline, on request
		
	Alpha-galactosidase A activity and genotype	If not previously performed or determined

**Kidney**	Serum creatinine, ionogram, BUN; morning spot urine for urinary protein/creatinine ratio and albumin/creatinine ratio Urinary Gb_3 _(optional)	Baseline. Every 3 months if CKD stage 1 or 2 and >1 g/day of proteinuria or CKD stage 4 Every 6 months if CKD stage 3 Every 12 months if CKD stage 1 or 2 and <1 g/day of proteinuria

**Cardiac**	Palpitations, angina Blood pressure, rhythm	Baseline, every 6 months Every evaluation visit
		
	ECG, echocardiography 2-D with Doppler	Baseline, every 12 months
		
	Holter monitoring	If an arrhythmia is suspected or palpitations are present
		
	Cardiac MRI	Every other year
		
	Coronary angiography	If clinical signs of angina

**Neurologic**	Acroparesthesias, fatigue, fever, heat and cold tolerance, stroke-related symptoms, TIA	Baseline, every 12 months
		
	Neurologic examination, questionnaires (Brief Pain Inventory)	Baseline, every 12 months
		
	Brain MRI without contrast	Baseline At time of a TIA or stroke event In females to document CNS involvement Every 3 years
		
	Magnetic resonance angiography	If cerebral vasculopathy should be excluded
		
	Comorbid stroke risk factors: Cholesterol (Total, LDL, HDL), triglycerides, Lpa, total plasma homocysteine	Baseline, every 12-24 months

**ENT**	Tinnitus, hearing loss, vertigo, dizziness	Baseline, every 6 months
		
	Audiometry, tympanometry, otoacoustic emissions	Baseline, every 12 months thereafter

**Ophthalmologic**	General ophthalmologic exam (slit-lamp, direct ophthalmoscopy, best corrected visual acuity, visual fields)	Baseline, every 12-24 months

**Pulmonology**	Cough, exertional dyspnea, wheezing, exercise intolerance	Baseline, every 12 months
		
	Spirometry	If clinical signs

**Gastrointestinal**	Postprandial abdominal pain, bloating, diarrhea, nausea, vomiting, early satiety, difficulty gaining weight Endoscopic evaluations	Baseline, every 12 months If symptoms persist or worsen despite treatment

**Skeletal**	Bone mineral density, 25(OH) vitamin D levels	Baseline

CKD stages: 1: GFR > 90 mL/min/1.73 m^2^; 2: 60<GFR < 89 mL/min/1.73 m^2^; 3: 30<GFR < 59 mL/min/1.73 m^2^; 4: 15<GFR < 29 mL/min/1.73 m^2^; 5: GFR < 15 = mL/min/1.73 m^2 ^or end stage renal disease (ESRD) (dialysis or transplantation).

##### Gastrointestinal symptoms

Gastrointestinal problems resulting from delayed gastric emptying and slow bowel movements may respond to metoclopramide [307] and changes in eating habits, e.g. small and frequent meals. Some success has been achieved by managing dyspepsia with H-2 blockers [51].

##### Skin symptoms

Laser methods to treat angiokeratomas have not shown good results in FD and are not able to prevent the formation of new lesions [57].

##### Cochleo-vestibular symptoms

Moderate hearing loss can be managed with hearing aids while profound deafness requires cochlear implants [51,137]. Vertigo-related nausea can be addressed with trimethobenzamide or prochlorperazine [51].

##### Renal function

FD is often associated with proteinuric chronic kidney disease, and it appears that the treatment paradigms that have proven to be effective in diabetes mellitus and other forms of proteinuric renal disease are also effective in FD [308]. The use of angiotensin-converting enzyme inhibitors (ACEi) or angiotensin receptor blockers (ARBs) is useful in patients with proteinuria (Table 3) [299]. Furthermore, these agents may help to control hypertension when present. Indeed, severe proteinuria does not respond to ERT alone [309], but carefully titrated ACEi/ARB therapy may be effective in lowering proteinuria [299,310,311]. In a pilot study, sustained reductions in proteinuria with stabilization of kidney function were achieved in a small number of patients with severe Fabry nephropathy receiving a combination of agalsidase beta at 1 mg/kg every other week (EOW) and ACEi/ARB therapy [312].

Although FD represents an interesting example of progressive proteinuric renal disease in which the usual blood pressure is lower than in other renal diseases, hypertension can occur and, if present [313], should be treated appropriately. Many patients with FD and renal involvement will require dialysis [314] and/or renal transplant [315,316]. Transplanted kidneys remain free of Gb_3 _accumulation and 5-year organ survival is above average for renal transplants [315-318].

##### Cerebrovascular involvement

The use of enteric coated aspirin for prophylaxis to minimize the risk of stroke is recommended in guidelines proposed by clinical experts [51]. Clopidrogel will be considered if aspirin is not tolerated and a combination of both drugs may be proposed in case of stroke or transient ischemic attack. Coumadin is often given to patients who have had stroke on aspirin and clopidrogel. Adequate intake of vitamins B12, B9, and B6 should be promoted [51] especially in case of hyperhomocysteinemia [277]. Statins may have potential beneficial effects [319].

##### Cardiac involvement

In the case of exertion chest pain, conventional anti-anginal therapy should be administered (calcium channel blockers that do not limit heart rate may be preferred to **β**-blockers as the later can aggravate both sinus bradycardia and the fact that some patients have a propensity to develop atrioventricular [AV] block). Beta-blockers are not necessarily contraindicated but should be used cautiously. Aspirin can be prescribed in case of isolated left atrial enlargement and warfarin treatment should be offered to any patient affected with FD and atrial fibrillation. Cardiac pacing or implantation of cardioverter defibrillator (ICD) devices is increasingly used in patients with FD with AV block or to prevent sudden cardiac death due to sustained ventricular tachycardia and malignant arrhythmia [81]. Amiodarone interferes with lysosome metabolism and should therefore probably be avoided during enzyme replacement. If there is evidence of heart failure, ACEi, ARBs or diuretics should be preferred to **β**-blockers because of the aforementioned caveats [88]. In patients with advanced congestive heart failure, heart transplantation is an option [207,320]. Vitamin D levels and lipid profil should be controlled and, if anormal, normalized, using both diet and statins for the later (Table 3).

##### Respiratory involvement

Cessation of smoking should be encouraged [299].

##### Endocrine dysfunction

Adequate monitoring of endocrine glands and hormonal therapy, when required, have to be performed in cases of subclinical endocrine dysfunction [171].

##### Bone involvement

Although no data exist, the use of biphosphonate therapy is currently being investigated. Vitamin D insufficiency or deficiency should also be corrected.

##### Psychological aspects

Psychological support should be provided. Anxiety and depression should be treated [159,161].

#### B. Prophylactic measures

**Figure 28:**
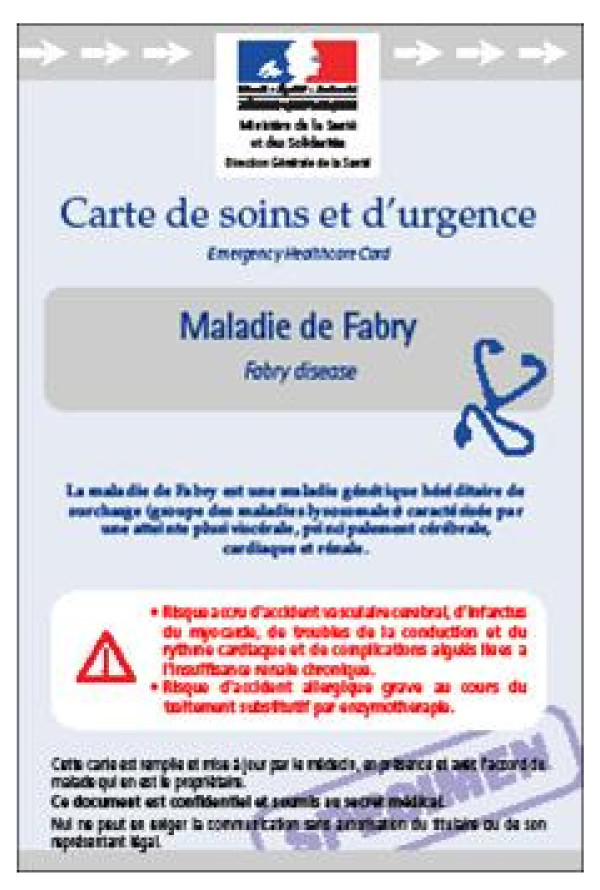
**Emergency healthcare card from the French Ministry of Health**: an emergency healthcare card was created by the Ministry of Health, the center of excellence for Fabry disease and patients' associations for Fabry disease or lysosomal storage diseases. The card is made of two parts: one of which contains general data on FD while the second one includes the personal medical history and medications of the patient in order to provide useful information for emergency care situations.

Patients should be advised to carry with them a letter and/or an emergency healthcare card (Figure 28) indicating the nature of their illness, the complications to which they are at risk, their current medication and the contact details of a medical practitioner. Intense physical activity and excessive sun exposure are inadvisable. Various medications such as chloroquine or amiodarone interfere with lysosome metabolism and their prescription is contraindicated in the license of recombinant **α**-galactosidase A (agalsidase alfa and agalsidase beta), and should therefore be avoided during enzyme replacement.

### XI - Enzyme replacement therapy

Conventional treatment does not address the underlying defect of FD and the year 2001 witnessed the introduction of ERT using recombinant human **α**-galactosidase A. Since then, long term safety and efficacy of replacement therapy have been investigated and ERT has been validated as a disease-specific therapeutic agent for patients affected with FD but with this, has come the realization that numerous aspects have yet to be explored and understood. As an example, current guidelines for starting ERT in patients vary from one country to another and remain a matter of debate especially in heterozygous females and children. Current expert recommendations [51] are presented in Table 4, but may evolve in the future. In Europe, there are currently two commercially available enzyme preparations for FD [321,322]: agalsidase alfa (Replagal^®^; Shire, Cambridge, MA, USA), produced using cultured human skin fibroblasts and registered for use at a dose of 0.2 mg/kg biweekly, and agalsidase beta (Fabrazyme^®^; Genzyme Corp, Cambridge, MA, USA), produced by the expression of human **α**-galactosidase cDNA in Chinese Hamster Ovary (CHO) cells and registered for a use at 1.0 mg/kg biweekly. The safety and efficacy of both enzymes have been assessed in randomized, double-blind, placebo-controlled trials [323-326] and their extension studies for agalsidase alfa [327,328] and agalsidase beta [309,329], studies originating from industry-sponsored registries [330-333] and investigator-sponsored studies independent from the industry [334-338]. Hereunder, we review the clinical efficacy data currently available for each drug since their marketing authorization within the European Union [339,340].

**Table 4:** Current proposed guidelines for starting enzyme replacement therapy in Fabry disease patients

Subpopulation	Guidelines for onset of ERT
Adult males (over 16 years)	At time of diagnosis of Fabry disease

Boys	At time of development of significant symptoms or if asymptomatic, consider at 7-10 years

Females (all ages)	Symptoms or evidence of progression of organ involvement

Note: the recommendations are proposed guidelines from an international panel of physicians with expertise in Fabry disease [51]. They are currently not followed in every country and may be revised when additional studies on the outcome of the safety and efficacy of enzyme replacement therapy become available.

#### A. Efficacy and safety data of agalsidase alfa treatment

Agalsidase alfa (Replagal^®^; Shire, Cambridge, MA, USA) is an enzyme replacement therapy for FD. Agalsidase alfa first received marketing authorization in the European Union in August 2001, and is approved for the treatment of FD in 45 countries. Agalsidase alfa is purified from a stably transfected human cell line and is infused at a dose of 0.2 mg/kg of body weight over a period of 40 minutes every 14 days [294,341]. Double-blind, randomized clinical trials of ERT with agalsidase alfa in FD involved relatively small numbers of patients [324,326] and most of the data presented here originates from industry-sponsored FOS^® ^or open-label clinical trials.

##### Amelioration of early clinical symptoms

In two pediatric clinical trials of ERT with agalsidase alfa, including 37 children [342,343], boys demonstrated reductions in plasma Gb_3 _levels, and both boys and girls reported reductions in neuropathic pain and in the use of neuropathic pain medications. Heart rate variability, which is classically reduced in boys with FD, was statistically significantly improved with 6 months of agalsidase alfa treatment [342-344]. With the possible exception of clearance in younger patients, agalsidase alpha appears to have comparable pharmacokinetic and pharmacodynamic profiles in pediatric and adult Fabry patients of both genders [345]. In the 3.5-year extension study of one of the pediatric clinical trials, there were sustained, statistically significant improvements in the clinical features of FD, including reduced plasma Gb_3 _levels, reduced pain severity assessed by the brief pain inventory (BPI) questionnaire, and improved heart rate variability. Mean urine Gb_3 _levels were reduced to normal range. Kidney function and left ventricular mass indexed to height remained stable throughout the study [346].

In a small open-label study, improvements in acroparesthesia and anhidrosis were associated with a normalization of sympathetic skin responses after 2 years on agalsidase alfa [347].

In a larger cohort of patients from the FOS^® ^observational database, pain severity was significantly reduced in 81 patients on agalsidase alfa for 2 years and in 62 patients on agalsidase alfa for 3 years, and all dimensions of pain perception were improved [43]. Improvements in health-related quality of life (QoL) paralleled improvements in pain and were maintained after 24 months of ERT [330,331].

In an analysis of agalsidase alfa replacement therapy in patients with FD who were enrolled in the FOS^®^, a clinically significant reduction of pain (defined as improvement of >1 point on the BPI) was recorded for average and worst pain (60.4% and 53.1% of patients, respectively) after 5 years of treatment [333]. Before initiation of ERT, QoL was worse in patients with FD than in the general population. Mean QoL deviation score from normal EuroQol values improved significantly compared with baseline after 5 years of treatment [from - 0.24 (0.30) to - 0.17 (0.28) p = 0.0483] [333].

There are several reports suggesting that ERT with agalsidase alfa may ameliorate the abdominal pain and diarrhea associated with FD. A reduction in the incidence of GI pain in 62 patients was shown after 12 months of ERT (from 49% to 39% of patients) and in 58 patients after 24 months of ERT [50]. The prevalence of diarrhea was also reduced after 12 and 24 months of ERT compared with baseline, the absence of a control group being a limitation of this study [50].

##### Renal function

In several studies, the estimated glomerular filtration rate (eGFR) and creatinine clearance remained stable after 1-2 years of ERT [330,332,348]. However, in a study aiming to determine the effects of ERT with agalsidase alpha on renal function in patients with Fabry nephropathy, eGFR declined in males with stage 1 and 2 kidney disease treated by agalsidase alfa at 0.2 mg/kg during 3 years, although proteinuria was under 1 g/24 h in all patients enrolled in this open-label study [349].

In patients whose renal function continues to decline while receiving agalsidase alfa at 0.2 mg/kg (eGFR decline of ≥ 5 mL/min/1.73 m^2^/year), there may be benefits from doubling the dose through weekly infusions rather than infusions every 2 weeks (mean rate of change in eGFR improved from - 8.0 ml/min/1.73 m^2^/year to - 3.3 ml/min/1.73 m^2^/year; *p *= 0.01) [328].

A recent meta-analysis showed that ERT with agalsidase alfa appears to slow down the decline in GFR in patients with mild to moderate nephropathy and baseline proteinuria under 1 g per day [350]. Patients with more advanced nephropathy and/or overt proteinuria did not respond as well to agalsidase alfa alone. No histological data was shown with respect to clearance of Gb_3 _from podocytes or other renal cell types. Treatment with agalsidase alfa did not improve proteinuria [350].

##### Cardiac morphology and function

In an open-label study, significant reductions in left ventricular mass (LVM) were observed in heterozygous women after 27 weeks on agalsidase alfa [351]. Mean ventricular wall thickness and LVM were reduced in a larger cohort of patients from the FOS^® ^after 1 and 2 years of ERT [330]. Of note, the largest decreases in LVM were observed in patients with the greatest degree of hypertrophy at baseline [330], a result that contrasts with those from a number of studies with agalsidase beta [20,91,338,352]. In a double-blind randomized clinical trial on a small number of patients with FD and cardiac involvement, ERT resulted in a progressive decrease in LVM measured by MRI (p = 0.041) after 6 months on agalsidase alfa at 0.2 mg/kg every other week [326]. Cardiomyocyte Gb_3 _clearance which was the primary efficacy endpoint did not reach statistical significance [326].

In an analysis of agalsidase alfa replacement therapy in patients with FD who were enrolled in the FOS^®^, treatment resulted in a sustained reduction in LVM index from 71.4 g/m^2.7 ^(SD 22.5) to 64.1 g/m^2.7 ^(SD 18.7) after 5 years (p = 0.0111) and a significant increase in midwall fractional shortening from 14.3% (SD = 2.3) to 16.0% (SD = 3.8) after 3 years (p = 0.02) [333]. Sentinel clinical cardiac and cerebrovascular events occurred in a greater proportion of patients with LVH than without LVH after 5 years of treatment [333].

##### Cerebrovascular events

Initial results on the effect of agalsidase alfa (0.2 mg/kg every other week) on CNS involvement in FD showed progression of white matter lesions in 2 out of 7 patients [128]. This study involved a small number of patients with a limited follow-up for 1 year [128] and, to date, it is not known if agalsidase alfa therapy can reduce or prevent the cerebrovascular complications and hearing loss associated with FD [353]. During the 4.5 year follow-up study of the original phase III pivotal trial, four out of the 25 patients (16%) suffered a cerebrovascular accident or a transient ischemic attack [327].

##### Severity score and causes of death

*S*core index of FD severity, such as the Mainz Severity Score Index (MSSI) [354], have shown a general reduction in disease severity after one year of ERT with agalsidase alfa [355,356].

Data on causes of death in a cohort of 1453 patients (699 male and 754 female) from 19 countries worldwide enrolled in the FOS^® ^were analysed, while causes of death of their affected relatives were analysed separately. The principal causes of death among 181 affected relatives of patients in FOS^®^, most of who had died before 2001, were renal failure in males (42%) and cerebrovascular disease in females (25%). In contrast, of the 42 patients enrolled in the FOS^® ^whose deaths were reported between 2001 and 2007, cardiac disease was the main cause of death in both male (34%) and female (57%) patients [87].

#### B. Efficacy and safety data of agalsidase beta treatment

Agalsidase beta (Fabrazyme^®^, Genzyme Corporation, Cambridge, MA, USA) is indicated for long-term ERT in patients with a confirmed diagnosis of FD. It is intended to replace deficient endogenous **α**-galactosidase A in these patients. World-wide, agalsidase beta is currently approved in 55 countries, including the USA. In February 2008, the European Medicine Agency's Committee for Medicinal Products for Human Use (CHMP) granted full marketing authorization to Fabrazyme^® ^superseding its approval under exceptional circumstances [Fabrazyme^® ^Summary of Product Characteristics (SPC)] [339].

##### Clearance of Gb_3 _from renal cells, urine and cardiac cells

Renal capillary endothelial cells were (nearly) completely cleared of Gb_3 _after 20 weeks of agalsidase beta at 1 mg/kg EOW in 98% of the patients in the original multicenter, randomized, placebo-controlled, double blind phase III clinical trial [323]. Complete clearance of Gb_3 _was also observed in mesangial and interstitial cells in the majority of patients [357]. All improvements were maintained with sustained treatment over 4.5 years, and signs of contained improvement in clearance from epithelial cells (podocytes, distal tubular epithelial cells) were noted, although Gb_3 _was never completely cleared from podocytes [309]. Reductions were less complete in non-capillary smooth muscle cells. The capacity of agalsidase beta at 1 mg/kg EOW to normalize Gb_3 _content of renal capillary endothelial cells after 20 weeks of treatment was confirmed in a bridging study in 13 Japanese male patients [358]. Urinary Gb_3 _excretion was reduced in both studies after 20 weeks of therapy [323,358].

In the heart, 5 months of agalsidase beta treatment in the phase III clinical trial [323] resulted in complete clearance of Gb_3 _from the microvasculature in 72% of treated patients compared with only 3% of placebo-treated patients (p < 0.001) [359]. The placebo group achieved similar results after 6 months of treatment in the open-label extension study [359]. In addition, the capillary endothelium remained free of Gb_3 _for up to 60 months [309] in 6 of 8 patients who consented to an end-of-study cardiac biopsy [359]. No clearance of Gb_3 _was observed in the cardiomyocytes during the trial [359].

Of note, repeated infusions with agalsidase beta over a prolonged period did not appreciably clear storage material in cells other than vascular endothelial cells in two case reports [360,361]. In the samples from the heart and some other tissues biopsied from two male patients after several months of ERT with agalsidase beta, only the endothelial cells were free of Gb_3 _and persistent storage was found in cardiomyocytes, smooth muscle cells, fibroblasts and sweat glands [361]. Similarly, extensive glycolipid storage deposits were seen in all organ systems with the exception of vascular endothelial cells in the autopsy study of a 47-year-old male patient who died after 2.5 years of ERT with agalsidase beta [360].

##### Amelioration of early clinical symptoms

Fourteen boys and 2 girls, 8 to 16 years old, were treated in an open-label pediatric clinical trial. A 12-week-observation period to collect baseline data preceeded the 48-week-treatment period when agalsidase beta (1 mg/kg) was infused intravenously at 1 mg/kg EOW. No primary efficacy endpoint was specified [362]. Before treatment, results of skin biopsies from 12 male patients showed moderate or severe Gb_3 _accumulation in superficial dermal capillary endothelial cells; with treatment, these cells were completely cleared of Gb_3 _in week-24 biopsies from all 12 male patients and in all available week-48 biopsies. Agalsidase beta was generally well tolerated; most treatment-related adverse events were mild or moderate with infusion-associated reactions involving rigors, fever, or rhinitis. Children treated with agalsidase beta experienced less pain and gastrointestinal problems, and were reported to have more energy and improved school attendance as documented by patients' diaries [362]. No overall significant change in serum creatinine, mild proteinuria and eGFR was found in pediatric patients after 48 weeks of treatment [362].

In the extension study of the original phase III trial in adult patients, pain scores as measured by the McGill Pain Questionnaire improved over time with sustained agalsidase beta treatment at 1 mg/kg EOW for those who reported pain at baseline and use of pain medications was reduced in some patients [309,329]. For most SF-36 components, patients experienced a mean improvement after long-term treatment with agalsidase beta (Fabrazyme^®^). The mean changes from pretreatment through month 54 for the components of Physical Functioning, Role Emotional, Body Pain, and Standardized Physical Component Scale (for patients with score <100 at first measurement before treatment) were statistically significant (*p *< 0.015, 0.031, 0.003, and 0.006, respectively) [309].

Pain reduction was also found in 2 other studies after ~20 months of agalsidase beta therapy at 1 mg/kg EOW [334,363]. One of these studies evaluated nerve fiber function in 22 males with Fabry neuropathy and reported subclass-dependent improvements in small nerve fiber function [363]. Such improvements were not seen in patients with severe thermal perception dysfunction at baseline [363].

Health-related quality of life was also measured using the SF-36^® ^health survey in 71 men and 59 women enrolled in the Fabry Registry^® ^who were treated with agalsidase beta and who had baseline and at least 2 yearly post-treatment health-related quality of life measurements. Long-term treatment with agalsidase beta resulted in substantial improvements in health-related quality of life in both men and women [364].

##### Renal function

**Figure 29:**
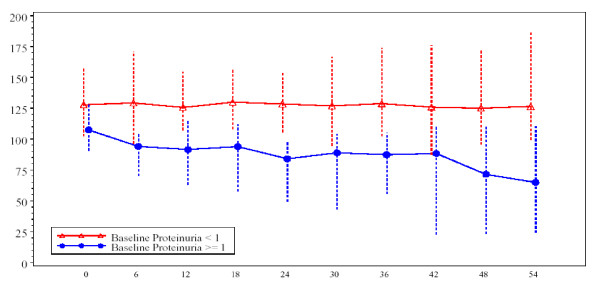
**Median estimated glomerular filtration rate (eGFR; ml/min per 1.73 m2) over time in 44 patients treated with agalsidase beta for 54 months:** Patients in the “as treated” population maintained a stable median eGFR during the 54-month treatment. Subgroup analyses of patients who were stratified by baseline proteinuria (>1 g/24 h versus <1 g/24 h) showed differences in the rate of eGFR decline during the 54-mo treatment period [309]. High (>1 g/24 h) baseline proteinuria was associated with higher rate of eGFR decline and increased probability of renal events.

**Figure 30:**
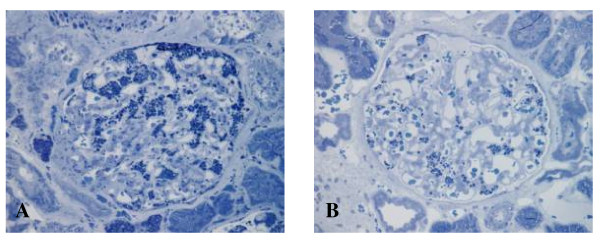
**Long-term agalsidase beta therapy decreases Gb_3 _accumulation in podocytes**: A) Kidney biopsy which was obtained prior to agalsidase beta therapy shows dark-staining granules in podocytes. B) By month 54, fewer Gb_3 _inclusions are evident from a specimen which was obtained from the same patient. Methylene blue/azure II stain, magnification × 400 [309].

It has been shown that renal function in adult patients can be preserved with sustained treatment with agalsidase beta at 1 mg/kg EOW [309]. Estimated glomerular filtration rate, proteinuria and serum creatinine remained stable and normal in the vast majority of patients treated for 4.5 years (54 months) [309]. The 6 patients showing a rise in serum creatinine shared a common profile at baseline including age >40 (n = 4/6), high proteinuria levels (> 2 g/24 h, n = 4/6) and significant glomerulosclerosis (> 50%, n = 4/4). This profile predisposed them to progression of renal disease, even under agalsidase beta therapy. The mean rate of eGFR decline for the remaining patients (n = 52) as a group was 0.4 ml/min per 1.73 m2/yr and not significantly different from 0 (p = 0.6785). Subgroup analyses were performed to examine the impact of baseline proteinuria or glomerulosclerosis on renal function during the study period. The mean yearly decline in eGFR in patients (n = 42) with low (< 1 g/24 h) proteinuria at baseline was minimal [mean eGFR slope = -1.0 ml/min per 1.73 m^2^/yr (1.0; p = 0.3052)] (Figure 29) [309], and not statistically different from normal yearly reduction of GFR [365]. Progressive Gb_3 _clearance from podocytes was observed on kidney biopsies (n = 8) after 54 months of ERT (Figure 30) [309].

Favorable renal outcomes in patients with less impaired renal function have also been reported [334]. Patients with normal kidney function (GFR > 90 ml/min/1.73 m^2^) at baseline treated for a mean of 23 months showed stabilization of kidney function, whereas patients with GFR < 90 ml/min/1.73 m^2 ^had a significant decrease in mean eGFR (from 71 to 60 ml/min/1.73 m^2^) [334].

##### Cardiac morphology and function

Several research groups have found reductions in LVH and amelioration of LV stiffness and regional myocardial function in patients with FD treated with agalsidase beta at 1 mg/kg EOW. Significant improvement in LV hypertrophy and function (both radial and longitudinal LV function) were found in a strain rate imaging study in 16 adult patients (mean age 42) treated for 1 year [91].

In another study, the effects of agalsidase beta (1 mg/kg EOW) on cardiac morphology, function, and late gadolinium enhancement were studied during 12 months of ERT. Only patients without late enhancement (LE) at baseline had significant reductions in LVM during ERT. No patients without late enhancement at baseline developed LE during ERT. Echocardiography revealed an improvement of regional myocardial function in patients without LE. In contrast, in patients with LE at baseline, the amount of LE significantly increased and the follow-up examinations showed neither regression of LVM nor improvement in regional myocardial function [20].

In an open-label study, stabilization of mean LVM has been demonstrated after 1 year of agalsidase beta treatment at 1 mg/kg EOW in patients aged >30 years who had significant degrees of baseline myocardial hypertrophy [335]. In contrast, neither resting or dipyridamole-stimulated myocardial perfusion nor myocardial perfusion reserve changed during ERT [335]. Self-estimated cardiovascular condition, QoL, diastolic function, exercise capacity, ECG parameters, ejection fraction and ventricular mass did not change in an open-label prospective follow-up study of 24-month ERT with agalsidase beta at 1 mg/kg EOW in 5 male and 4 female patients. ERT had only minimal effect on symptoms and cardiovascular morphology and function [366].

In an open-label study on 11 patients (8 males and 3 females) a significant reduction in myocardial T2 relaxation times was noted in all myocardial regions on MRI, (interventricular septum, apex, and lateral wall) after a mean treatment duration of 45 months with agalsidase beta at 1 mg/kg EOW [367].

##### Progression to major renal, cardiac, or cerebrovascular events, or death

A multicentric, double-blind, randomized, placebo-controlled phase IV study has shown that agalsidase beta at 1 mg/kg EOW can slow the progression of the serious, life-threatening complications of FD, even in patients who already have overt kidney dysfunction [325]. This study enrolled 82 patients (72 males, 10 females; aged 20-72 years) who were followed for 18.5 months (median). The group of 51 patients randomized to receive agalsidase beta treatment, overall, had pronounced renal disease with a mean eGFR of 53 mL/min/1.73 m^2 ^at baseline. A significant 61% reduction of the risk of progression to major renal, cardiac, or cerebrovascular events, or death, was found in treated patients as compared to placebo-treated patients in the per-protocol analysis that adjusted for an imbalance in baseline proteinuria [hazard ratio, 0.39 (CI, 0.16 to 0.93); p = 0.034]. Greater and highly significant treatment effects were seen in patients that had less severe renal impairment at baseline (eGFR > 55 ml/min/1.73 m^2^) [325].

To date, it is not known if agalsidase beta therapy can reduce or prevent the cerebrovascular complications and hearing loss associated with FD [309,329]. In the long-term extension study of the original pivotal trial, five of 58 patients (9%) experienced symptomatic stroke or transient ischemic attacks as an adverse event [309].

Whether a lower dose could maintain the Gb_3 _clearance achieved with 1.0 mg/kg was explored in a study where 21 adult male patients were treated with agalsidase beta for 6 months at 1.0 mg/kg EOW followed by 18 months at 0.3 mg/kg/2 weeks. A lower dose of agalsidase beta was sufficient in some, but not all, patients to maintain the cellular Gb_3 _clearance achieved with 1.0 mg/kg/2 weeks. Long-term clinical effects of transitioning to the lower dose have not been evaluated [368].

#### C. Comparison between agalsidase alfa and agalsidase beta treatments

##### Randomized controlled trials

The results of the published randomized controlled clinical trials and their extension studies, together with the pediatric trials for the two enzyme preparations, agalsidase alfa [324,326,327,342] and agalsidase beta [309,323,329,359,362] are shown in Table 5.

**Table 5:** Comparison of safety and efficacy outcomes of the enzyme replacement therapies

	Fabrazyme^®^, agalsidase beta - 1 mg/kg/14 days	Replagal^®^, agalsidase alfa - 0.2 mg/kg/14 days
**Efficacy data on renal histology**	At 6 months (n = 58): - Total clearance of Gb_3 _in renal intersticial capillary endothelial cells [323] - Total clearance of Gb_3 _in glomerular, mesangial and interstitial cells [323] - Partial clearance of Gb_3 _in arterial smooth muscular cells [357] At 54 months: - Significant clearance maintained in several renal cells types (n = 8) [309]	At 6 months (n = 26), glomeruli with mesangial widening decreased by a mean of 12.5% for patients receiving agalsidase alfa *versus *a 16.5% increase for placebo (p = 0.01) [324]

**Efficacy data on renal function**	Significant risk reduction (-61%) of renal, cardiac, cerebrovascular complications and death in *per protocol *analysis that adjusted on an imbalance in baseline proteinuria (n = 74; p = 0.034) [325] At 54 months (n = 44) [309]: Stabilization of eGFR for 90% of patients (- 0,4 ml/min/1,73 m^2^/year) (n = 42)	At 6 months (n = 26) [324]: - Significant increase of creatinine clearance in treated group *versus *placebo - No significant difference of inuline clearance between the 2 groups At 54 months (n = 25) [327]: - Patients with stage 1 CKD: average eGFR loss of - 1,6 ml/min/1,73 m^2^/year - Patients with stage 2 CKD: average eGFR loss of - 2,6 ml/min/1,73 m^2^/year - Patients with stage 3 CKD: average eGFR loss of - 4,9 ml/min/1,73 m^2^/year [327] At 5 years (FOS^® ^data): - Male patients with stage 1 CKD: mean yearly fall in eGFR = -2.83 ml/min/1,73 m^2^/year - Male patients with stage 2 CKD: mean yearly fall in eGFR = -2.17 ml/min/1,73 m^2^/year - Male patients with stage 3 CKD: mean yearly fall in eGFR = -3.0 ml/min/1,73 m^2^/year [333]

**Efficacy data on cardiac histology**	Significant Gb_3 _clearance in cardiac endothelial cells at 6 months [323] maintained at 54 months [309,359] No clearance of Gb_3 _in cardiomyocytes [359]	A mean 20% reduction in myocardial Gb_3 _content was demonstrated over the 6 months of ERT compared to a mean 10% increase in patients receiving placebo (p = 0.42) [326]

**Efficacy data on cardiac function and geometry (clinical trials)**	Significant risk reduction (-61%) of renal, cardiac, cerebrovascular complications and death in the *per protocol *analysis that adjusted on an imbalance in baseline proteinuria (n = 74; p = 0.034) [325]	Left ventricular mass, as measured by MRI, was significantly reduced following 6 months of treatment with agalsidase alfa compared with placebo (p = 0.041) [326]

**Efficacy data on peripheral nervous system based on clinical trials**	Significant improvement in pain scores at 54 months (p = 0.016) [309] Significant improvement in quality of life at 54 months (p = 0,007) (n = 52) [309]	Significant decrease of average pain scores at 6 months (n = 26) [324]

**Efficacy data on pediatric population based on clinical trials**	At 12 months (n = 16): - Significant clearance of plasma Gb_3 _(normalization) - Significant clearance of Gb_3 _in skin specimens - Patient diaries documented significant reductions in school absences due to sickness. - Reduction in gastro-intestinal symptoms [362]	Enzyme replacement with agalsidase alfa was safe. The exploratory efficacy analysis documented increased clearance of Gb_3_, reductions in neuropathic pain and in the use of pain medication, and improvement of autonomic function (n = 24) [342,346]

**Immunogenicity**	IgG reported for 90% of patients [323] IgE reported in a few patients [295,376]	IgG reported for 56% of the patients No report of IgE

**Infusion time**	90 (once safety established) - 180 minutes	40 - 60 minutes

**Home based treatment availability after hospital initiation**	Yes	Yes

**Treatment costs (in France)**	- Vial cost (35 mg): 3,370 euros - Annual cost of therapy for a 70 kg adult patient: 161,781 € (year 2010)	- Vial cost (3.5 mg): 1,685 euros - Annual cost of therapy for a 70 kg adult patient: 161,781 € (year 2010)

**Market authorization approval**	- European market authorization approval: August 2001 - European Medicines Agency (EMA) exceptional circumstances lifted (February 2008) - American market authorization approval: April 2003	- European market authorization approval: August 2001 - European Medicines Agency (EMA) exceptional circumstances maintained - American market authorization approval: none

Note: the comparison has been limited to randomized placebo-controlled clinical trials, their extension studies, and paediatric trials.

##### Head to head clinical trials

The efficacy of and tolerability towards the two agalsidase preparations administered at identical protein dose (0.2 mg/kg/14 days) were compared in a randomized controlled open-label trial. The study revealed no difference in reduction of LVM or other disease parameters after 12 and 24 months of treatment with either agalsidase alfa (Replagal^®^) or beta (Fabrazyme^®^) at a dose of 0.2 mg/kg biweekly. Treatment failure occurred frequently in both groups and seemed related to age and severe pre-treatment disease [336]. In another comparative study, the occurrence of **α**-galactosidase A antibodies and their effect on urinary and plasma Gb_3_, chitotriosidase and clinical outcome were assessed in 52 patients after 12 months of treatment with either 0.2 mg/kg agalsidase alpha (10 males, 8 females) or beta (8 males, 5 females) or 1.0 mg/kg agalsidase beta (10 males, 11 females) [337]. Alpha-galactosidase A antibodies frequently developped in male patients (18/28) and interfered with urinary Gb_3 _excretion. From urinary Gb_3 _studies, it appears that persistence of antibodies impairs ERT at a dose of 0.2 mg/kg EOW. Infusion of a dose of 1.0 mg/kg resulted in a more robust decline in Gb_3_, less impact of antibodies, stable renal function and reduction of LVM [337]. Some concerns have been expressed about the methodological design and data interpretation of the later study [369].

An independent study of patients with FD in Canada, aiming to compare the effects of agalsidase alfa, 0.2 mg/kg/14 days, *versus *agalsidase beta, 1.0 mg/kg/14 days on clinical outcomes is currently ongoing [370]. However, interpretation of the study results will be meaningful only if any imbalance between the agalsidase alfa and agalsidase beta treated groups in terms of baseline parameters is corrected, and proper analysis of the data accounts for confounding factors such as gender of patients and nature of clinical events.

The supply of agalsidase beta has been reduced, since June 2009, due to production problems. The supply shortage of agalsidase beta resulted in some patients either being switched to receiving agalsidase alfa or to having a reduced dose of agalsidase beta. The potential impact of IgG antibodies on the response to enzyme replacement therapy in these patients remains unresolved [327,337,368,371,372]. Patients in whom the dose or formulation of ERT has been amended will require careful monitoring in order to assess impact on safety and clinical efficacy [27] and help decision making when supply of agalsidase beta is restored.

##### Kidney function

The goal for treatment of Fabry nephropathy is reduction in the rate of loss of GFR to <-1.0 mL/min/1.73 m^2^/year [77,373].

After 5 years of treatment with agalsidase alfa at the dose recommended by the manufacturer (0.2 mg/kg EOW), data reported in the FOS^® ^database showed that mean yearly fall in estimated GFR was - 2.83 mL/min/1.73 m^2 ^for male patients with chronic kidney disease (CKD) stage 1 at baseline [333], statistically different (p = 0.0001) from the normal yearly reduction of - 0.9 mL/min/1.73 m^2 ^[365]. The mean yearly loss of eGFR for men with stage 2 disease at baseline was - 2.17 mL/min/1.73 m^2 ^(p = 0.0004). In male patients with stage 3 CKD at baseline, the mean yearly fall in eGFR after 5 years was - 3.0 ml/min/1.73 m^2 ^(p = 0.006) [333,374]. In contrast, corresponding values for men with CKD stage 1 or stage 2 disease and proteinuria < 1 g/day at baseline treated for 5 years with agalsidase beta at 1 mg/kg EOW (n = 42) were - 1.005 mL/min/1.73 m^2^/year [309], not statistically different (p= 0.3052) from normal yearly reduction rate [365].

#### D. Practical considerations of ERT for Fabry disease

##### Infusion management

During the pivotal, double-blind trials of agalsidase alfa (Replagal^®^) [324] and agalsidase beta (Fabrazyme^®^) [323], 57% (8/14) and 59% (34/58) of patients experienced mild-to-moderate infusion-related reactions, respectively, the incidence peaking around the fifth to eighth infusion. Fevers, chills and rigors were the only treatment-related adverse events occurring significantly more frequently in the treatment group than in the placebo group; all were transient, mild-to-moderate in severity and were managed conservatively [323]. In the follow-up study with agalsidase alfa, 13 of 25 patients experienced an infusion-reaction during or shortly after one or more infusions. These reactions typically consisted of facial flushing and rigors [327]. After 3 to 5 years of treatment, the number of patients treated with agalsidase beta experiencing infusion reactions fell to between 10 and 20%, suggesting that patients develop tolerance to the infusions over time [309,329].

The precise cause of the infusion-associated reactions is unknown, but may be related to IgG antibodies specific to the infused enzyme (IgG seroconversion occurred in 24% of agalsidase alfa treated-patients [339,340] and in 51 of the 58 (88%) who received agalsidase beta during double-blind [323] or open-label treatment [329]), or to complement activation.

Experience gained in our center [375] suggests that infusion-associated reactions (IAR) tend to occur during the first 6 months of treatment - usually after 20-40 minutes of the infusion - and last for approximately 10-30 minutes. The risk of events tends to increase with increasing infusion rates. Based on these observations, it is recommended that, at the first occurrence of an infusion reaction, the patient's temperature and vital signs should be assessed and the infusion rate temporarily slowed or stopped. In the case of a severe reaction, the infusion must be stopped and the administration of antihistamines and/or corticosteroids should be considered. The infusion can be continued in the case of mild reactions, with close supervision. After cessation of or a decrease in symptoms, the infusion may be re-started and the infusion rate gradually increased to the original rate. Subsequent infusions should be started at a lower infusion rate and increased every 30 minutes. Pre-medication with an antihistamine, paracetamol and/or dexamethasone (1 hour before infusion) may also be considered [295].

**Figure 31:**
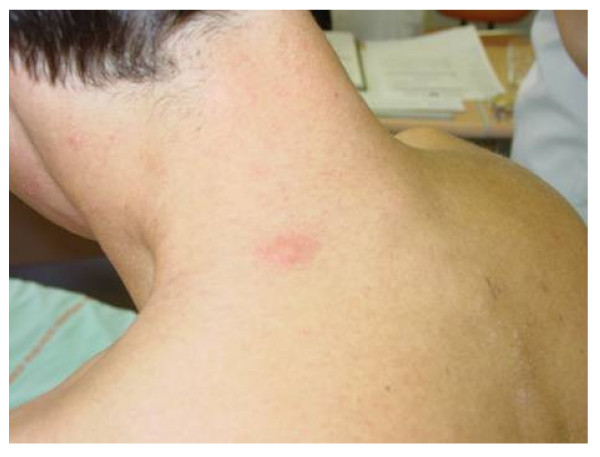
**Skin rash during infusion of recombinant α-galactosidase A in a patient with positive IgE antibodies to agalsidase beta**: In year 2002, a 39-year-old male Fabry patient (*GLA *mutation p.Ala121Pro) was initially treated with agalsidase beta (1 mg/kg EOW). ERT was changed to agalsidase alfa (0.2 mg/kg EOW) after 18 months due to poor tolerance (mild laryngeal edema, urticaria and chills during infusions). Two years later, a rash appeared on both arms during agalsidase alfa infusions. In 2007, concomittant deterioration of kidney function on agalsidase alfa (mGFR decreased from 85 to 70 mL/min/1.73 m^2^) led to switch ERT back to agalsidase beta. No data was obtained with respect to antibodies (IgG or IgE) to agalsidase alfa. After 1 year of agalsidase beta therapy, extensive skin rash and bronchospasm appeared during the infusions despite premedication (hydroxyzine, paracetamol and oral steroids) and minimal infusion rates (0.05 - 0.2 mg/min) and kidney function kept on deteriorating (mGFR = 54 mL/min/1.73 m^2^). The patient tested positive for IgE to agalsidase beta and ERT was discontinued. Mutation p.Ala121Pro is not responsive to the ASSC deoxygalactonojirymicin [424]. Both rechallenge protocol and concomitant use of immunosuppressive therapy and ERT are currently being considered.

A few of the approximately 3000 patients treated to date with agalsidase beta have developed plasma IgE antibodies and a few others have had a positive prick-test together with urticaria or skin rash (Figure 31). Most patients have successfully undergone a rechallenge protocol [376]. No IgE antibodies have been detected during agalsidase alfa treatment [327]. Whether seroconversion affects treatment efficacy is currently unknown but neutralizing antibodies to both agalsidase alfa and agalsidase beta have been demonstrated [371] and shown to lead to a relapse in urinary [327,336,377] and cutaneous [372] Gb_3_. This warrants further studies since in Gaucher disease [378], another lysosomal storage disorder due to the deficient activity of acid **β**-glucosidase [379], neutralizing antibodies have been shown to block the catalytic activity of the exogeneous enzyme and lead to a deterioration of clinical course in the very rare instances where they occur [380]. In these cases, the potential use of immunosuppressive therapy in combination with ERT should be investigated [381].

##### Infusion during dialysis and post transplant

Many physicians involved in treating patients with FD using ERT have queried whether dialysis influences the pharmacokinetics of the recombinant enzyme. Although experience of infusing the enzyme during dialysis is currently limited, no problems have been encountered to date. Virtually no difference in the plasma activity of agalsidase beta was found regardless of whether or not the infusion was given during hemodialysis [382]. The procedure used a low-flux polysulphone filter, with which there was no loss of enzyme. Theoretically, enzyme adsorption to the filter could occur. A recommendation, therefore, is to begin enzyme infusion approximately 15 minutes after the start of dialysis, by which time the membrane's surface will be covered by plasma proteins such as fibrinogen or albumin, reducing the likelihood of enzyme adsorption to the membrane and to the tubing system. The feasibility of infusing ERT during dialysis confers a considerable practical advantage to patients requiring dialysis [382].

##### Pregnancy

Although ERT is theoretically contra-indicated during pregnancy and lactation, both agalsidase alfa [383,384] and agalsidase beta [385,386] have been used in a limited number of cases. No adverse event was reported and both recombinant enzymes appear safe. However, few data are available and the decision to initiate or maintain ERT during pregnancy should be made on an individual basis and carefully monitored [385].

##### Home therapy

Home therapy can help to alleviate the burden of intravenous infusions every 14 days for stable patients who tolerate the infusions and have a suitable home environment [387]. Several reports suggest that patients appreciate home treatment and, if implemented successfully, ERT can be administered in the home setting in a safe and reliable manner [310,327,388]. This should however not lead to decreased medical care and patients should be referred to a tertiary center of excellence every 6 to 12 months.

### XII - Prognosis

With age, progressive damage to vital organ systems develops and at some point, organs may start to fail in functioning. End-stage renal disease and life-threatening cardiovascular or cerebrovascular complications limit life-expectancy of untreated males and females to approximately 50 and 70 years, representing reductions of 20 and 10 years, respectively, as compared to the general population [25,26]. While it is hoped that long-term enzyme therapy can halt disease progression, the importance of adjunctive therapies should be noted and the possibility of developing an oral therapy drives forward research into active site specific chaperones.

### XIII - Current research

#### A. Basic research: cellular model of Fabry disease

In a recent study, a cell model of FD was established [389]. The expression of **α**-galactosidase A was transiently silenced by RNA interference in HK2 and primary human renal epithelial cells and stably silenced in HK2 cells by retroviral transfection with small hairpin RNA (shRNA). All of the silenced cells had reduced viability, significant accumulation of intracellular Gb_3_, and a modest but significant increase in membranous Gb_3 _(CD77) expression compared to non-silenced cells. When silenced HK2 cells were reconstituted with agalsidase alfa, they decreased their membranous CD77 expression to levels indistinguishable from those of non-silenced cells. These data suggest that membranous CD77 levels may mirror Gb_3 _tissue load and that CD77 expression levels may be used to monitor the efficacy of ERT [390].

#### B. Basic research: animal models

Genetically authentic animal models of human lysosomal diseases occur spontaneously in many mammalian species. However, most are among larger domestic or farm animals with only few well-defined genetic lysosomal diseases known among rodents. This status changed dramatically with the advent of the combined homologous recombination and embryonic stem cell technology, which allows directed generation of mouse models that are genetically equivalent to human diseases [391]. This technology has allowed generation of knock-out mice for FD [392,393] as well as transgenic mice [394,395]. These animal models have played an important role in studies of the pathogenesis [15,396,397] and treatments (including bone marrow transplant [398], substrate deprivation [399], enzyme replacement therapy [322,377,400], active site specific chaperones [401] and gene therapy [402-407]) for FD. While the utility of these mouse models is obvious, species differences in metabolic pathways must always be remembered, if the ultimate goal of the study is application to human patients.

#### C. Clinical research: registries and outcome surveys

The Fabry Registry^® ^[408] and the Fabry Outcome Survey^® ^(FOS^®^) [409] are ongoing, observational databases that compile clinical and laboratory data on patients with FD. As of March 2010, the Fabry Registry^® ^and FOS^® ^included 3200 and 1700 patients respectively. All patients with FD are eligible for enrollment in the Fabry Registry^®^, regardless of age, gender, symptoms, or whether they are receiving ERT while enrollment in FOS^® ^is limited to patients treated with agalsidase alfa or naïve to ERT. Patient and physician participation is voluntary. All patients provide informed consent through local institutional review boards/ethics committees and may decline to participate or withdraw consent at any time. Treating physicians determine the actual frequency of assessments according to patients' individualized needs. A schedule of recommended clinical assessments is available in the Fabry Registry^® ^[408]. Given the voluntary nature of reporting data, patients' ages at clinical assessments and time intervals between assessments are variable. Due to the rarity of the condition, clinical trials of ERT in FD generally involved relatively small numbers of patients and much of the available data on the natural history of the disease and the long term safety and efficacy of the recombinant enzymes available in the literature stems from the FOS^® ^[40,43,53,84,87,144,293,330,331,410] or the Fabry Registry^® ^[23,24,51,76,111,184,411,412].

### XIV - Future perspectives

#### A. Use of a modified alpha-N-acetylgalactosaminidase in the development of enzyme replacement therapy for Fabry disease

The human lysosomal enzymes alpha-galactosidase (**α**-gal A, EC 3.2.1.22) and alpha-N-acetylgalactosaminidase (**α**-NAGAL, EC 3.2.1.49) share 46% amino acid sequence identity and have similar folds. The active sites of the two enzymes share 11 of 13 amino acids, differing only where they interact with the 2-position of the substrates. Using a rational protein engineering approach, the enzymatic specificity of **α**-galactosidase A and **α**-NAGAL were interconverted. The engineered **α**-NAGAL [or **α**-NAGAL(EL)] retains the antigenicity of **α**-NAGAL but has acquired the enzymatic specificity of the **α**-galactosidase A enzyme. Comparison of the crystal structures of the designed enzyme to the wild-type enzymes shows that active sites of **α**-galactosidase A and **α**-NAGAL superimpose well, indicating success of the rational design. The designed enzymes might be useful as non-immunogenic alternatives in ERT for treatment of FD [413].

In another experiment, a modified alpha-N-acetylgalactosaminidase (NAGA or **α**-NAGAL, EC 3.2.1.49) with **α**-galactosidase A-like substrate specificity was designed on the basis of structural studies and was produced in CHO cells. The enzyme acquired the ability to catalyze the degradation of 4-MU-alpha-D-galactopyranoside. There was no immunological cross-reactivity between the modified NAGA and **α**-galactosidase A, and the modified NAGA did not react to serum from a patient with FD treated with recombinant **α**-galactosidase A. The enzyme cleaved Gb_3 _accumulated in cultured fibroblasts from a patient with FD. Furthermore, like recombinant agalsidases currently used for ERT for FD, the enzyme injected intravenously into FD model mice prevented Gb_3 _storage in the liver, kidneys, and heart and improved the pathological changes in these organs. Because the modified NAGA is not expected to cause an allergic reaction in patients with FD, it is promising as a new and safe enzyme for ERT [414].

#### B. Active site specific chaperones

**Figure 32:**
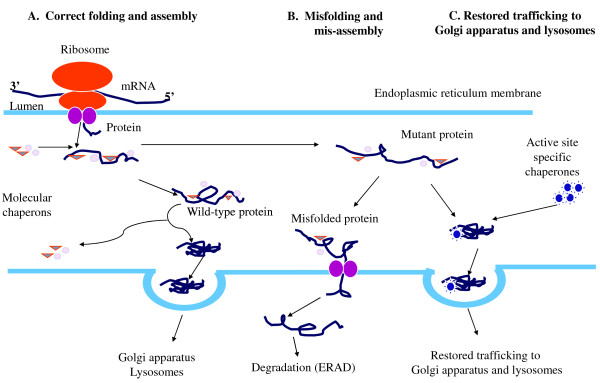
**Proposed mechanism of action of active site-specific chaperones (ASSCs)**: A: During synthesis of a wild-type lysosomal enzyme, cells assemble amino acids in a correctly folded tertiary structure. Molecular chaperones are naturally occurring molecules that assist in protein folding. B: In contrast, mutant misfolded lysosomal enzymes are unstable and retained in the endoplasmic reticulum (ER) where they may not meet quality control and are prone to endoplasmic reticulum-associated degradation (ERAD). C: ASSCs are designed to stabilize and rescue misfolded lysosomal enzymes, leading to reduced ER retention or accumulation, and enhanced trafficking to the Golgi apparatus and the lysosome where they dissociate from the enzyme.

In FD, a significant number of disease-causing mutations are missense mutations, which cause the newly synthesized lysosomal protein to be unstable, but still catalytically competent [231,415]. Despite the fact that unstable mutant **α**-galactosidases are catalytically comparable to their wild type counterpart in their purified forms [204], the newly synthesized enzymes are unable to undergo trafficking to their appropriate location within the cell - the lysosomal compartment (Figure 32). Studies of trafficking and degradation of various mutant forms of **α**-galactosidase A indicate that the mutant enzymes are retained in the endoplasmic reticulum (ER) and degraded by ER-associated degradation (ERAD) because of their misfolded conformations [204]. This provides a rationale for a therapeutic intervention using active-site-specific chaperones to stabilize the conformation or reduce misfolding of the mutant protein in order to prevent the premature degradation by ERAD (Figure 32) [416-419].

Enzyme inhibitors from the imino-sugars family were shown to be effective active-site-specific chaperones, causing an increase in residual enzyme activity and stabilizing enzyme activity in cultured lymphoblasts and transfected COS-1 cells [420,421]. Subsequently, galactose, a weak inhibitor of **α**-galactosidase A, was intravenously infused to a male patient with the cardiac variant of FD at a dose of 1 g/kg body weight every other day. After 3 months of galactose infusions, the myocardial fibres, which initially appeared severely hypertrophic and extensively vacuolated were smaller, and vacuolization was decreased [422].

The imino sugars are monosaccharide mimetics, characterized by having a nitrogen atom in place of the ring oxygen present in monosaccharides and often are potent inhibitors of glycosidases. As the imino sugars have a high affinity for the active site of the target enzyme, they can also act as active-site-specific chaperones, assisting protein folding or stabilizing misfolded enzymes [423]. 1-Deoxygalactonojirymicin (DGJ), currently under investigation by the trade name of Amigal™ (migalastat hydrochloride; Amicus Therapeutics, Cranbury, NJ, USA), is a small imino sugar which mimics the **α**-galactose of Gb_3_, the substrate for α-galactosidase A, when it binds to the active site of the enzyme. The firm binding between DGJ and the mutant enzyme shifts the folding and stability of the enzyme in favor of the appropriate and proper conformation, potentially permitting a smooth escape from the ER for further maturation and trafficking to the lysosomal compartment [419] (Figure 32). DGJ may be effective only in patients with specific, "responsive" *GLA *mutations coding for a mutant **α**-galactosidase with enhancable residual enzyme activity [419,424]. DGJ is an orally active, small molecule drug which could provide additional advantages of convenience and cost savings. However, since DGJ is primarily an inhibitor of **α**-galactosidase A activity, finding the right dosing and regimen for chaperoning is a key issue of this novel therapeutic approach [419]. Phase II extension and phase III clinical trials are ongoing.

## List of abbreviations

5' UTR: 5' untranslated region; **α**-gal A: alpha-galactosidase A; **α**-NAGAL (NAGA): alpha-N-acetylgalactosaminidase; ACE: angiotensin-converting enzyme; ACEi: angiotensin-converting enzyme inhibitors; ARBs: angiotensin receptor blockers; ASSC: active site specific chaperone; BPI: Brief Pain Inventory; CCA: common carotid artery; CHO: Chinese hamster ovary; CKD: chronic kidney disease; CNS: central nervous system; CT: computed tomography; DGJ: deoxygalactonojirymicin; CHMP: European Medicines Agency's Committee for Medicinal Products for Human Use; eGFR: estimated glomerular filtration rate; EMA: European Medicines Agency; EOW: every other week; ER: endoplasmic reticulum; ERAD: Endoplasmic Reticulum associated degradation; ERT: enzyme replacement therapy; FD: Fabry disease; FDA: Food and Drug Administration; FOS^®^: Fabry Outcome Survey^®^; Gb_3_: globotriaosylceramide; GFR: glomerular filtration rate; GI: gastro-intestinal; IAR: infusion-associated reaction; ICD: implantation of cardioverter defibrillator; IMT: intima-media thickness; IRB: Institutional Review Board; LE: late enhancement; LSD: lysosomal storage diseases; LVH: left ventricular hypertrophy; LVM: left ventricular mass; MRI: magnetic resonance imaging; MSSI: Mainz Severity Score Index; NSAID: non-steroidal anti-inflammatory drug; QoL: quality of life; RRT: renal replacement therapy; SD: standard deviation; SF-36: Short form with 36 items; SPC: Summary of Product Characteristics; TIA: transient ischemic attack.

## Competing interests

Dominique P. GERMAIN is a consultant for Genzyme Corporation and Shire HGT. He has received speaker's fees, research support and honoraria from Genzyme Corporation and Shire HGT.

## Appendix

### I - Organizations that provide support and information for patients with Fabry disease and their families

Fabry International Network (FIN)

http://www.fabryintnetwork.com

Fabry Support and Information Group (FSIG)

http://www.fabry.org

French Center of Excellence for Fabry disease

http://www.centre-geneo.com

### II - Web sites with medical, technical, and bibliographic information about Fabry disease and/or the *GLA *gene

*Orphanet*

The portal for rare diseases and orphan drugs

http://www.orpha.net

*The Human Gene Mutation Database *at the Institute of Medical Genetics in Cardiff

http://www.hgmd.cf.ac.uk/ac/index.php

*Online Mendelian Inheritance in Man (OMIM)*

A catalog of human genes and genetic disorders. The data base contains textual information, pictures, reference information, and links to NCBI's Entrez database of MEDLINE articles.

http://www.ncbi.nlm.nih.gov/omim

*ClinicalTrials.gov*

is a registry of federally and privately supported clinical trials conducted in the United States and around the world. ClinicalTrials.gov gives you information about purpose of a trial, who may participate, locations, and phone numbers for more details.

http://clinicaltrials.gov/ct2/results?term=fabry

### III - Fabry Disease Registries

*Fabry Registry*^®^

https://www.lsdregistry.net/fabryregistry/

*Fabry Outcome Survey*^® ^(*FOS*^®^)

http://www.globaloutcomesurveys.com
